# Endothelial TDP-43 controls sprouting angiogenesis and vascular barrier integrity, and its deletion triggers neuroinflammation

**DOI:** 10.1172/jci.insight.177819

**Published:** 2024-02-01

**Authors:** Víctor Arribas, Yara Onetti, Marina Ramiro-Pareta, Pilar Villacampa, Heike Beck, Mariona Alberola, Anna Esteve-Codina, Angelika Merkel, Markus Sperandio, Ofelia M. Martínez-Estrada, Bettina Schmid, Eloi Montanez

**Affiliations:** 1 Department of Physiological Sciences, Faculty of Medicine and Health Sciences, University of Barcelona and Bellvitge Biomedical Research Institute (IDIBELL), L’Hospitalet del Llobregat, Spain.; 2 Celltec-UB, Department of Cell Biology, Physiology, and Immunology, Faculty of Biology, and; 3 Institute of Biomedicine (IBUB), University of Barcelona, Barcelona, Spain.; 4 Walter Brendel Centre of Experimental Medicine, Biomedical Center, Institute of Cardiovascular Physiology and Pathophysiology, Ludwig-Maximilians-University Munich, Planegg-Martinsried, Germany.; 5 CNAG-CRG, Centre for Genomic Regulation, Barcelona Institute of Science and Technology, Barcelona, Spain.; 6 Josep Carreras Leukemia Research Institute (IJC), Barcelona, Spain.; 7 German Center for Neurodegenerative Diseases (DZNE), Munich, Germany.

**Keywords:** Angiogenesis, Vascular biology, Endothelial cells

## Abstract

TAR DNA-binding protein 43 (TDP-43) is a DNA/RNA-binding protein that regulates gene expression, and its malfunction in neurons has been causally associated with multiple neurodegenerative disorders. Although progress has been made in understanding the functions of TDP-43 in neurons, little is known about its roles in endothelial cells (ECs), angiogenesis, and vascular function. Using inducible EC-specific TDP-43–KO mice, we showed that TDP-43 is required for sprouting angiogenesis, vascular barrier integrity, and blood vessel stability. Postnatal EC-specific deletion of TDP-43 led to retinal hypovascularization due to defects in vessel sprouting associated with reduced EC proliferation and migration. In mature blood vessels, loss of TDP-43 disrupted the blood-brain barrier and triggered vascular degeneration. These vascular defects were associated with an inflammatory response in the CNS with activation of microglia and astrocytes. Mechanistically, deletion of TDP-43 disrupted the fibronectin matrix around sprouting vessels and reduced β-catenin signaling in ECs. Together, our results indicate that TDP-43 is essential for the formation of a stable and mature vasculature.

## Abstract 2

Endothelial DNA/RNA-binding protein TDP-43 is essential for formation of a stable and mature vasculature, and loss of TDP-43 results in vascular defects and inflammation.

## Graphical abstract

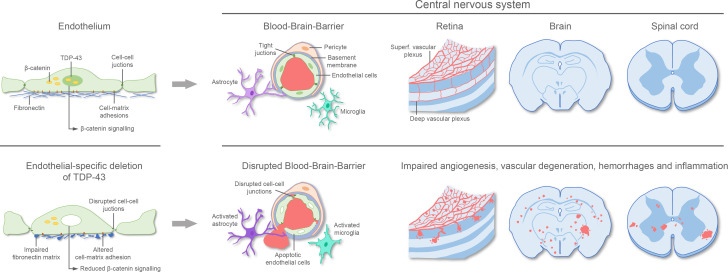

## Introduction

The function of the central nervous system (CNS) depends on the formation and integrity of a complex vascular network that ensures adequate supply of oxygen and nutrients (1). Developing CNS is vascularized through angiogenic invasion of blood vessels from vascular networks outside the CNS (2). Maturing CNS blood vessels establish highly selective semipermeable cellular membranes called blood-brain barrier (BBB), blood–spinal cord barrier (BSCB), and blood-retina barrier (BRB) that control the flow of molecules and cells from the systemic circulation to the CNS parenchyma (3–5). These blood-CNS barriers are essential for maintaining the internal milieu of the CNS necessary for proper neuronal function and for protecting the CNS parenchyma from toxins and pathogens present in the circulation. Defects in the integrity of these vascular barriers are associated with inflammatory and immune responses that can trigger neuronal loss and underlie the ontogeny or progression of several neurological disorders, such as stroke, diabetic retinopathy, and neurodegenerative diseases (6, 7). The functional unit underlying blood-CNS barriers is the neurovascular unit, which consists of a monolayer of endothelial cells (ECs), sealed by tight junctions (TJs) and resting on a basement membrane, surrounded by pericytes and astrocytes (8). CNS vascularization and blood-CNS barrier development are controlled by multiple signaling systems, including integrin-mediated cell-matrix adhesion signaling and Wnt/β-catenin signaling (9–12). Upon binding to the extracellular matrix (ECM), integrins recruit adaptor and signaling proteins to their cytoplasmic domains to form focal adhesions (FAs), through which they relay signals into the cells. Integrin-mediated signals promote EC proliferation and migration during vessel sprouting and the stabilization of the nascent vasculature (9–11). On the other hand, binding of Wnt to the Frizzled/Lrp receptor complex on ECs stabilizes β-catenin in the cytoplasm, promoting its translocation to the nucleus, where it activates the transcription of target genes involved in vascular growth and barrier formation (12).

TAR DNA-binding protein 43 (TDP-43) is a highly and ubiquitously expressed DNA/RNA-binding protein that shuttles between the nucleus and the cytoplasm to regulate different aspects of RNA metabolism including transcription, splicing, stabilization, and transport (13). Loss of TDP-43 results in incorrect splicing of pre-mRNAs, often disrupting their translation and promoting nonsense-mediated decay (14). Cytoplasmic aggregation of TDP-43, accompanied by its nuclear clearance, is a common pathological hallmark of several neurodegenerative diseases, including amyotrophic lateral sclerosis (ALS), frontotemporal dementia (FTD), Alzheimer’s disease, and limbic-predominant age-related TDP-43 encephalopathy (LATE) (15–17). Cytoplasmatic TDP-43 aggregation is thought to compromise its function in the nucleus, ultimately leading to neurodegeneration. The identification of missense mutations in the TDP-43 encoding gene (*Tardbp*) in patients with ALS, mechanistically linked neurodegeneration to TDP-43 function (18–20). Although neurons and glia cells have been the main focus of research on TDP-43–associated pathologies, cytoplasmic aggregation, and nuclear clearance of TDP-43 has also been detected in the CNS vasculature of ALS and patients with FTD (21), suggesting that TDP-43 function in CNS vascular cells of these patients may also be affected. Interestingly, LATE, one of the most prevalent TDP-43 proteinopathy, has been linked to cerebral small vessel pathology (22). However, little is known about the role of TDP-43 in ECs and vascular homeostasis.

TDP-43 binds and regulates the metabolism of nearly 30% of the entire transcriptome in many different cell types and organ systems (23, 24); consequently, constitutive deletion of the murine *Tardbp* gene leads to periimplantation lethality, long before the vasculature is formed (25–27). Similarly, constitutive loss of TDP-43 in zebrafish is embryonic lethal (28). The lethality of TDP-43–deficient zebrafish embryos is associated with abnormal blood vessel patterning, neuronal defects, and muscle degeneration (28). The vascular malformation phenotype is very severe and is the first visible phenotype in TDP-43–deficient embryos, suggesting that TDP-43 is critical for EC function and proper vessel formation. Consistent with these findings, we have recently reported that TDP-43 controls intersegmental vessel growth in zebrafish embryos (29). However, the role of endothelial TDP-43 in postnatal CNS angiogenesis and blood-CNS barrier function has not been studied so far and remains poorly understood.

In this study, using EC-specific TDP-43–KO mice, human and mouse TDP-43–deficient ECs, and RNA-Seq analysis, we demonstrate that TDP-43 is essential for retinal sprouting angiogenesis, vascular network remodeling, blood-CNS barrier integrity, and CNS blood vessel stability. Consequently, mice lacking endothelial TDP-43 exhibit multiple hemorrhages and vascular degeneration in the brain and spinal cord. These vascular defects are associated with an inflammatory response with activation of microglia and astrocytes. At the cellular level, we show that TDP-43 is essential for EC proliferation and survival, as well as for cell-ECM adhesion and cell-cell junction stability. Mechanistically, we show that TDP-43 regulates fibronectin (FN) matrix assembly during sprouting angiogenesis and β-catenin signaling in ECs.

Overall, our results uncover novel roles for TDP-43 in the growth and stabilization of CNS vessels, and they identify endothelial–TDP-43 as a potential contributing factor to the vascular disorders and inflammation observed in patients diagnosed with TDP-43–associated diseases.

## Results

### TDP-43 function in ECs is critical for retinal angiogenesis.

**Figure 1:**
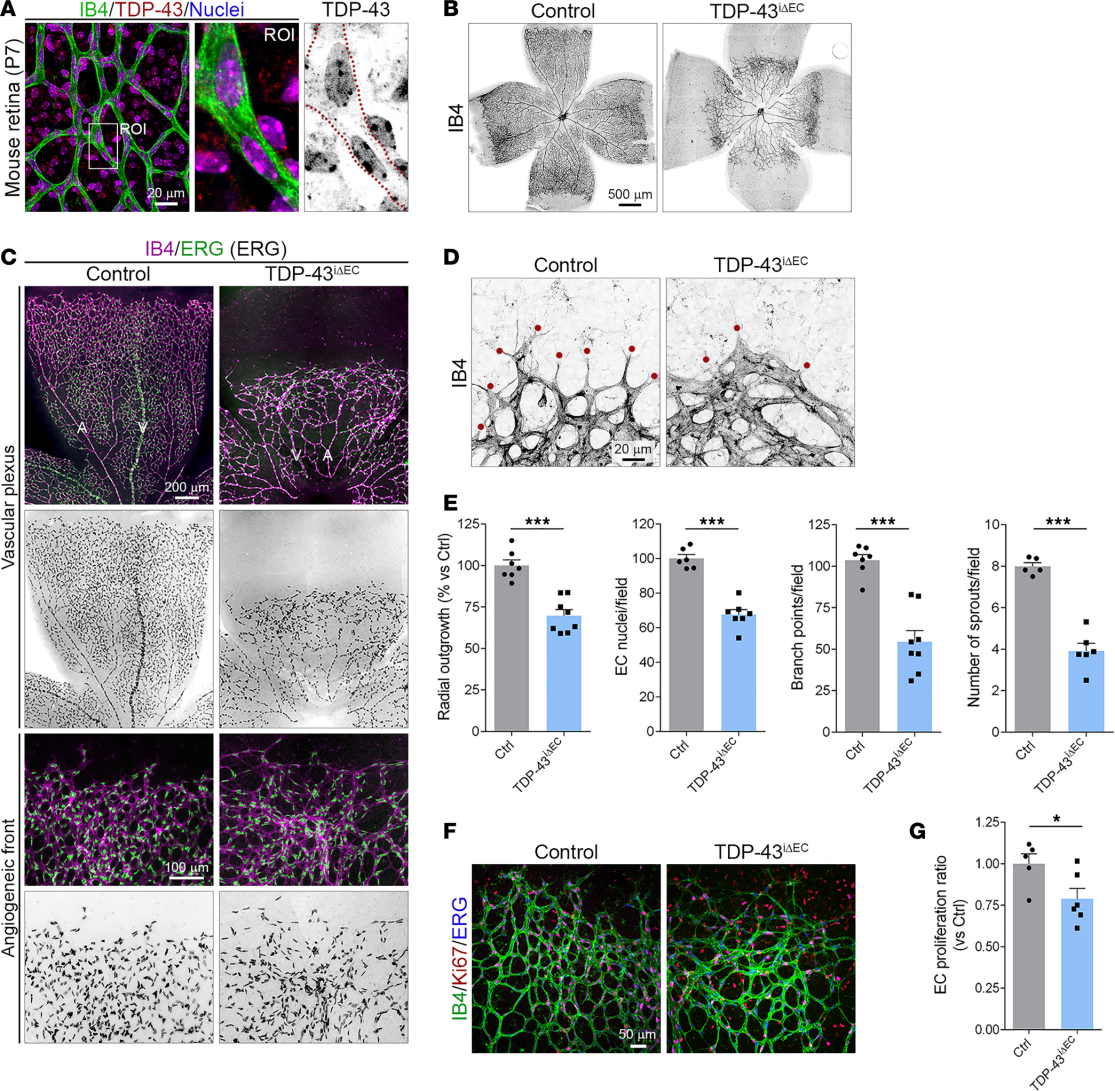
Postnatal endothelial cell–specific TDP-43 inactivation induces sprouting defects in the retina. (**A**) Confocal high-magnification image of P7 WT mouse retinas stained for the endothelial membrane marker isolectin B4 (IB4) (green), TDP-43 (red), and cell nuclei labeled with Hoechst (blue). Dotted red lines highlight the endothelial border of the blood vessels. White rectangle indicate the magnified region of interest (ROI). Scale bar: 20 μm. (**B**) Low-magnification overview images of whole-mount P7 control and *TDP-43*^i*Δ*EC^ retinas stained for IB4 to visualize vascularization and vascular outgrowth of the primary vascular plexus. Note reduction of vessel radial outgrowth in *TDP-43*^i*Δ*EC^ retinas compared with control retinas. Scale bar: 500 μm. (**C**) Confocal high-magnification images of control and *TDP-43*^i*Δ*EC^ P7 retinas stained for IB4 (majenta) and the endothelial cell (EC) nuclear marker ERG (green). Note the clustering of ECs at the vessel growth front of *TDP-43*^i*Δ*EC^ retinas. Arteries (A) and veins (V) are indicated. Scale bar: 200 μm. (**D**) Confocal high-magnification images of the angiogenic growth front of P7 control and *TDP-43*^i*Δ*EC^ retinas stained for IB4. Red dots indicate endothelial sprouts. (**E**) Quantification of vascular parameters in P7 control and *TDP-43*^i*Δ*EC^ retinas as indicated. Data are shown as mean ± SEM. ***P < 0.001. Mann-Whitney *U* test. (**F**) Confocal high-magnification images of control and *TDP-43*^i*Δ*EC^ P7 retinas stained for IB4 (green), the cell proliferation marker Ki67 (red) and ERG (blue). Scale bar: 50 μm. (**G**) Quantification of the relative ECs proliferation ratio. Data are shown as mean ± SEM. *P < 0.05. Mann-Whitney *U* test.

In healthy cells, TDP-43 localizes predominantly in the nucleus, where it controls mRNA processing (13). To determine whether TDP-43 is expressed in ECs of developing vessels, we analyzed TDP-43 protein expression pattern in retinas of P7 and P16 mice and in brain and liver sections of P16 mice, and we found that it is expressed in ECs in vivo and localizes mainly in the nucleus (Figure 1A and Supplemental Figure 1, A–C; supplemental material available online with this article; https://doi.org/10.1172/jci.insight.177819DS1). The analysis also showed that TDP-43 was evenly expressed throughout the entire vasculature, with clear expression in ECs of capillaries, arteries, and veins in the remodeling vascular plexus as well as in sprouting ECs at the vessel growth front (Supplemental Figure 1, A and B). Immunostaining of TDP-43 in cultured human umbilical vein ECs (HUVECs) showed that human ECs also express TDP-43 mainly in the nucleus (Supplemental Figure 1D). To uncover the function of TDP-43 in ECs, we crossed *TDP-43^fl/fl^* mice (30) with tamoxifen-inducible *Cdh5Cre^ERT2^* mice (31). Tamoxifen administration in newborn or 8-week-old mice resulted in sudden death of *TDP-43^fl/fl;Cdh5CreERT2^* (hereafter referred to as *TDP-43^iΔEC^*), but not of *TDP-43^fl/fl^* (control) mice, mice between 11 and 22 days after the first tamoxifen injection (Supplemental Figure 1E). These results indicate that endothelial TDP-43 is critical for vascular function.

Next, to uncover the role of endothelial TDP-43 in sprouting angiogenesis in vivo, we analyzed postnatal retinal vascularization. During the first postnatal week, a primary vascular plexus grows within the retinal ganglion layer (32). Following a FN template, blood vessels expand from the optic stalk toward the retinal periphery and establish a superficial vascular plexus around P8. Therefore, we induced TDP-43 gene deletion in EC in newborns mice via tamoxifen administration between P1 and P3, and we analyzed retinal vascularization at P7. Western blot analysis of the lung lysates from P6 *TDP-43^fl/fl^* and *TDP-43^iΔEC^* mice showed downregulation of TDP-43 expression when compared with lysates from Cre-negative control littermates (Supplemental Figure 2A). TDP-43 staining of P7 retinas confirmed a strong decrease in TDP-43 expression in ECs, but not in surrounding non-ECs, of *TDP-43^iΔEC^* mice (Supplemental Figure 2B). Isolectin B4 labeling of control and *TDP-43^iΔEC^* retinas showed delayed radial vessel outgrowth, decreased vessel branching, and reduced endothelial sprouting at the vessel growth front in *TDP-43^iΔEC^* retinas compared with control retinas (Figure 1, B–E, and Supplemental Figure 2C). Immunostaining of ERG, a specific nuclear marker of ECs, showed a decreased number of ECs in *TDP-43^iΔEC^* retinas compared with control retinas and irregular distribution of ECs at the angiogenic front of *TDP-43^iΔEC^* retinas (Figure 1, C and E). EC proliferation, assessed by Ki67 staining, decreased with the loss of TDP-43 (Figure 1, F and G). Together, these observations indicate that TDP-43 function in the ECs is required for endothelial sprouting and proliferation, and they argue for a role of TDP-43 in EC migration.

### Impaired endothelial FN matrix assembly in TDP-43^iΔEC^ retinas.

**Figure 2:**
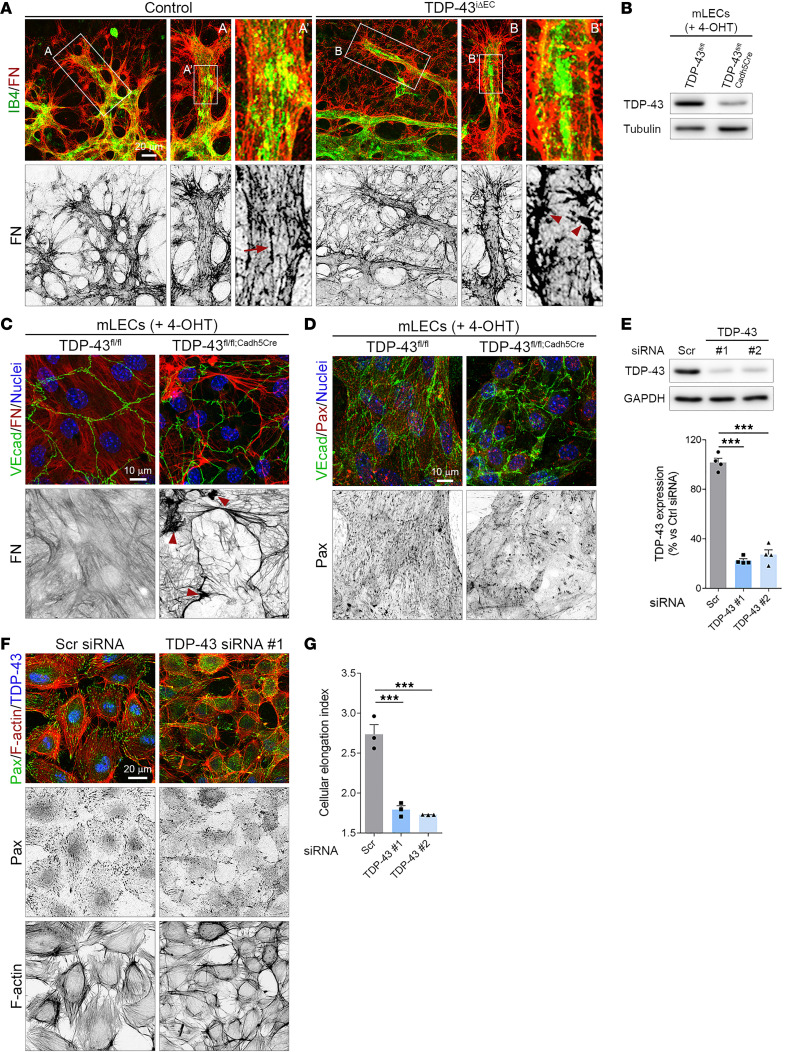
TDP-43 is required for FN matrix assembly and cell-matrix adhesion. (**A**) Confocal high-magnification images of P7 control and *TDP-43*^i*Δ*EC^ retinas stained for IB4 (green) and fibronectin (FN, red). White rectangles indicate magnified regions. Note the FN fibrils (arrows) associated to control vessels and the presence of irregular FN aggregates (arrowheads) in *TDP-43*^i*Δ*EC^ vessels. A indicates magnified region of image. Scale bar: 20 μm. (**B**) Western blot analysis of TDP-43 in total lysates of TDP-43^fl/fl^ and TDP-43^fl/fl;Cadh5Cre^ mouse lungs ECs (mLECs) 48 hours after 4-hydroxytamoxifen (4-OHT) treatment. Tubulin was used as a loading control. B indicates magnified region of image. (**C**) Confocal high-magnification images of TDP-43^fl/fl^ and TDP-43^fl/fl;Cadh5Cre^ mLECS stained for EC marker VE-cadherin (VEcad, green), FN (red), and Hoechst (nuclei, blue). Note the abnormal FN aggregates (arrowheads) in TDP-43^fl/fl;Cadh5Cre^ mLECs. Scale bar: 10 μm. (**D**) Confocal high-magnification images of TDP-43^fl/fl^ and TDP-43^fl/fl;Cadh5Cre^ mLECS stained for VEcad (green), the focal adhesion maker Paxillin (Pax, red), and Hoechst (nuclei, blue). Scale bar: 10 μm. (**E**) Western blot analysis of TDP-43 in total lysates of human umbilical vein ECs (HUVECs) transfected with either control (scramble, Scr) or TDP-43 small interfering RNAs (siRNAs). GAPDH was using as a loading control. Graph shows the quantification of the mean TDP-43 protein expression levels corrected for background and normalized to expression in Scr and TDP-43 siRNA–transfected HUVECs. Data are shown as mean ± SEM. ***P < 0.001. One-way ANOVA. (**F**) Confocal high-magnification images of control and TDP-43–depleted HUVECs stained for Pax (green), F-actin (red), and TDP-43 (blue). Scale bar: 20 μm. (**G**) Quantification of the ratio between the longest side and the shortest side (cellular elongation index) of the ECs in control and TDP-43–depleted HUVECs. Data are shown as mean ± SEM. ***P < 0.001. One-way ANOVA.

During retinal vascular development, ECs assemble a fibrillar matrix of FN that promotes vessel sprouting and branching (33). FN staining of P7 control and *TDP-43^iΔEC^* retinas showed severe morphological alterations in the FN matrix around the sprouting vessels of *TDP-43^iΔEC^* mice (Figure 2A). Whereas in control retinas, sprouting vessels were associated with a dense FN matrix, organized as a fibrillar meshwork with thin FN fibrils running longitudinally along the vessel axis, sprouting vessels of *TDP-43^iΔEC^* retinas were associated with a disorganized FN matrix, with sparse fibrils and multiple irregular aggregates of FN attached to the blood vessels (Figure 2A). During retinal angiogenesis, FN is mainly expressed by ECs and astrocytes (33, 34). To address whether FN matrix defects around *TDP-43^iΔEC^* vessels were consequence of impaired assembly of EC-derived FN, we isolated ECs from *TDP-43^fl/fl^* and *TDP-43^iΔEC^* mice, treated them with 4-hydroxytamoxifen (4-OHT), and assessed FN matrix assembly. Forty-eight hours after of 4-OHT treatment, *TDP-43^iΔEC^* cells showed decreased TDP-43 protein levels compared with control cells (Figure 2B). Similar to the situation in *TDP-43^iΔEC^* retinas, FN staining of control and TDP-43–depleted ECs showed disturbed FN matrix assembly in the absence of TDP-43 (Figure 2C). While control cells assembled a dense meshwork of thin FN fibrils, TDP-43–depleted cells assembled a disorganized and sparse FN matrix with thicker fibrils and irregular aggregates (Figure 2C). In addition, staining for paxillin, a marker of focal complexes and FAs, showed that FN matrix defects in TDP-43–deficient ECs correlated with reduced FA formation (Figure 2D). Next, to determine whether TDP-43 also regulates cell-matrix adhesion in human ECs, we knocked down TDP-43 protein expression in cultured HUVECs (Figure 2E). While control ECs showed multiple focal complexes along the membrane edges in the vicinity of FA, TDP-43–depleted ECs showed few focal complexes, and paxillin was detected mainly in aberrant matrix adhesion structures located at the cell periphery (Figure 2F). Morphological analysis showed reduced cell spreading of TDP-43–depleted ECs compared with control cells (Figure 2G). Taken together, these results indicate that deletion of TDP-43 impairs endothelial FN matrix assembly during sprouting angiogenesis and impaired EC-ECM adhesion.

### Endothelial TDP-43 is required for vascular remodeling and EC ingression into the deep retina.

**Figure 3:**
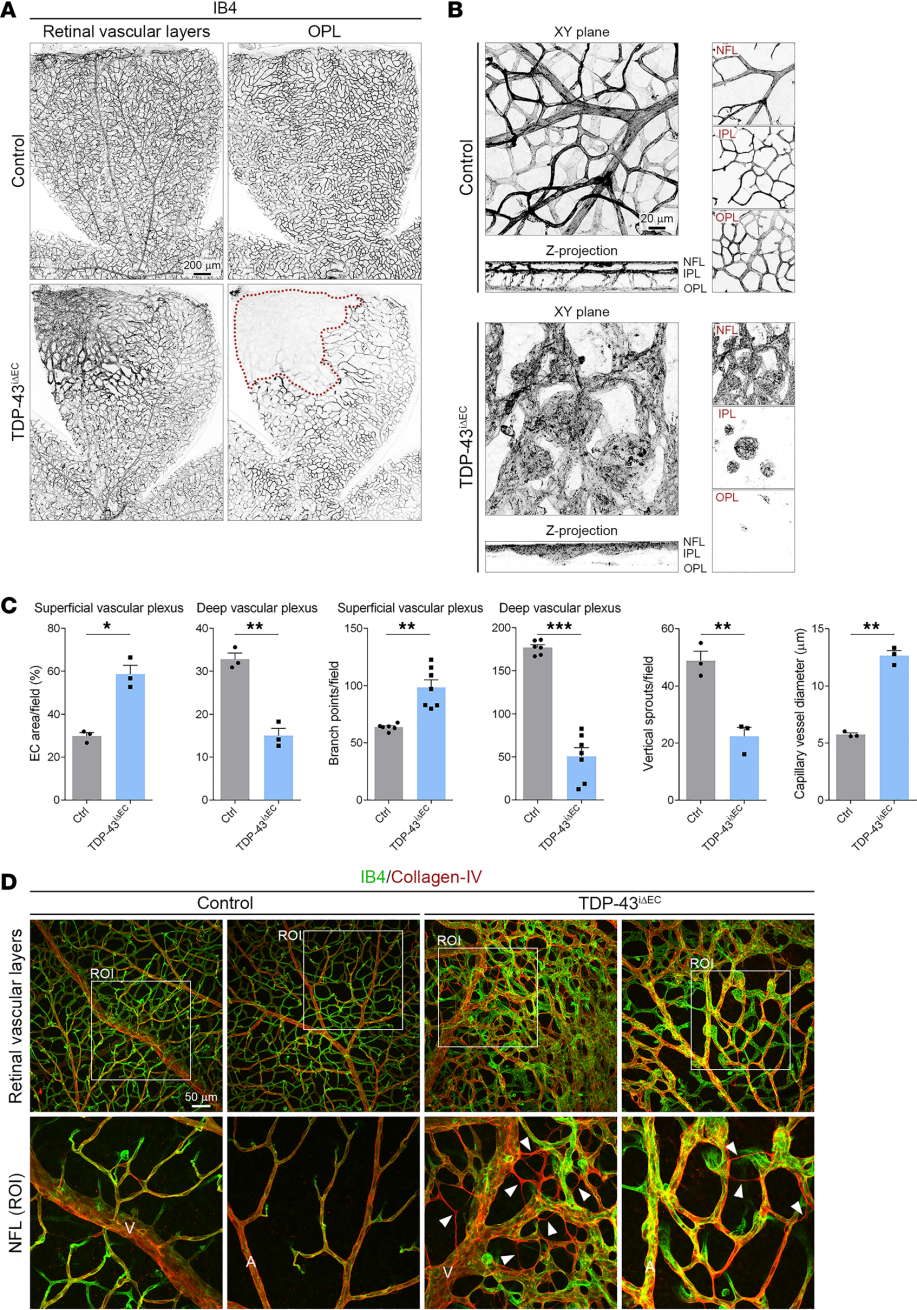
TDP-43 is indispensable for vessel remodeling and vascular plexus formation in the deeper retina. (**A**) Confocal high-magnification images of P16 control and *TDP-43*^i*Δ*EC^ retinas stained for IB4. Dotted line highlights the avascular area in the deep vascular plexus of *TDP-43*^i*Δ*EC^ retinas. Scale bar: 200 μm. (**B**) Optical sections of *Z*-stacked confocal high-magnification images were divided to illustrate the vascular plexus in the nerve fiber layer (NFL), inner plexiform layer (IPL), and outer plexiform layer (OPL). Note the bulging endothelial structures associated with the dilated vessels in *TDP-43*^i*Δ*EC^ retinas. Scale bar: 20 μm. (**C**) Quantification of vascular parameters in the control and *TDP-43*^i*Δ*EC^ P16 retinas as indicated. Data are shown as mean ± SEM. *P < 0.05, **P < 0.01, ***P < 0.001. Mann-Whitney *U* test. (**D**) Confocal high-magnification images of control and *TDP-43*^i*Δ*EC^ P16 retinas stained for IB4 (green) and collagen-IV (red). White squares indicate the magnified ROIs. Note the abundance of empty collagen-IV sleeves (arrowheads) in *TDP-43*^i*Δ*EC^ retinas. Arteries (A) and vein (V) are indicated. Scale bar: 50 μm.

After the formation of the superficial vascular plexus, endothelial sprouts descend vertically through the inner retina to form 2 additional capillary layers, the deep and intermediate vascular plexus (35). The deep vascular plexus develops in the outer plexiform layer (OPL) and reaches the periphery of the retina around P12, while the intermediate vascular plexus forms in the inner plexiform layer (IPL) between P12 and P15 (35). To further characterize the role of TDP-43 in retinal vessel growth, we induced the deletion of TDP-43 in ECs from P5 to P7 in *TDP-43^iΔEC^* mice and analyzed retinal vascularization at P16. The analysis revealed severe defects in deep retina vascularization in *TDP-43^iΔEC^* mice (Figure 3, A–C). *TDP-43^iΔEC^* retinas showed large avascular zones at the periphery of the OPL and numerous round aggregates of ECs in the vicinity of the superficial vascular plexus in the nerve fiber layer (NFL) (Figure 3, A and B, and Supplemental Figure 3A). In addition, vertical sprouts in the superficial vascular plexus, as well as vessel density and branching in the OPL, were reduced in *TDP-43^iΔEC^* retinas compared with control retinas (Figure 3C). From P9 onward and in parallel to the formation of vascular layers in the deep retina, the superficial vascular plexus undergoes extensive remodeling, eventually establishing an efficient and mature hierarchical vascular network (35). Superficial vascular plexus remodeling involves regression of excessive vessels, pruning of side branches, and reduction of vessel diameter (11). Quantification analysis showed increased vessel branching and capillary vessel diameter in the superficial vascular plexus of *TDP-43^iΔEC^* retinas compared with control retinas (Figure 3C). Regressing ECs leave empty basal membrane sleeves rich in collagen-IV (36). Staining of collagen-IV revealed abundant basement membrane segments devoid of ECs in *TDP-43^iΔEC^* retinas but not in control retinas, arguing that vessel remodeling is also perturbed in *TDP-43^iΔEC^* mice (Figure 3D and Supplemental Figure 3B). Together, these findings establish that endothelial TDP-43 is indispensable for endothelial sprouting into the deep retina and for the formation of a stable and mature vascular network.

### Endothelial deletion of TDP-43 impairs cell-cell junction morphology and BRB integrity.

**Figure 4:**
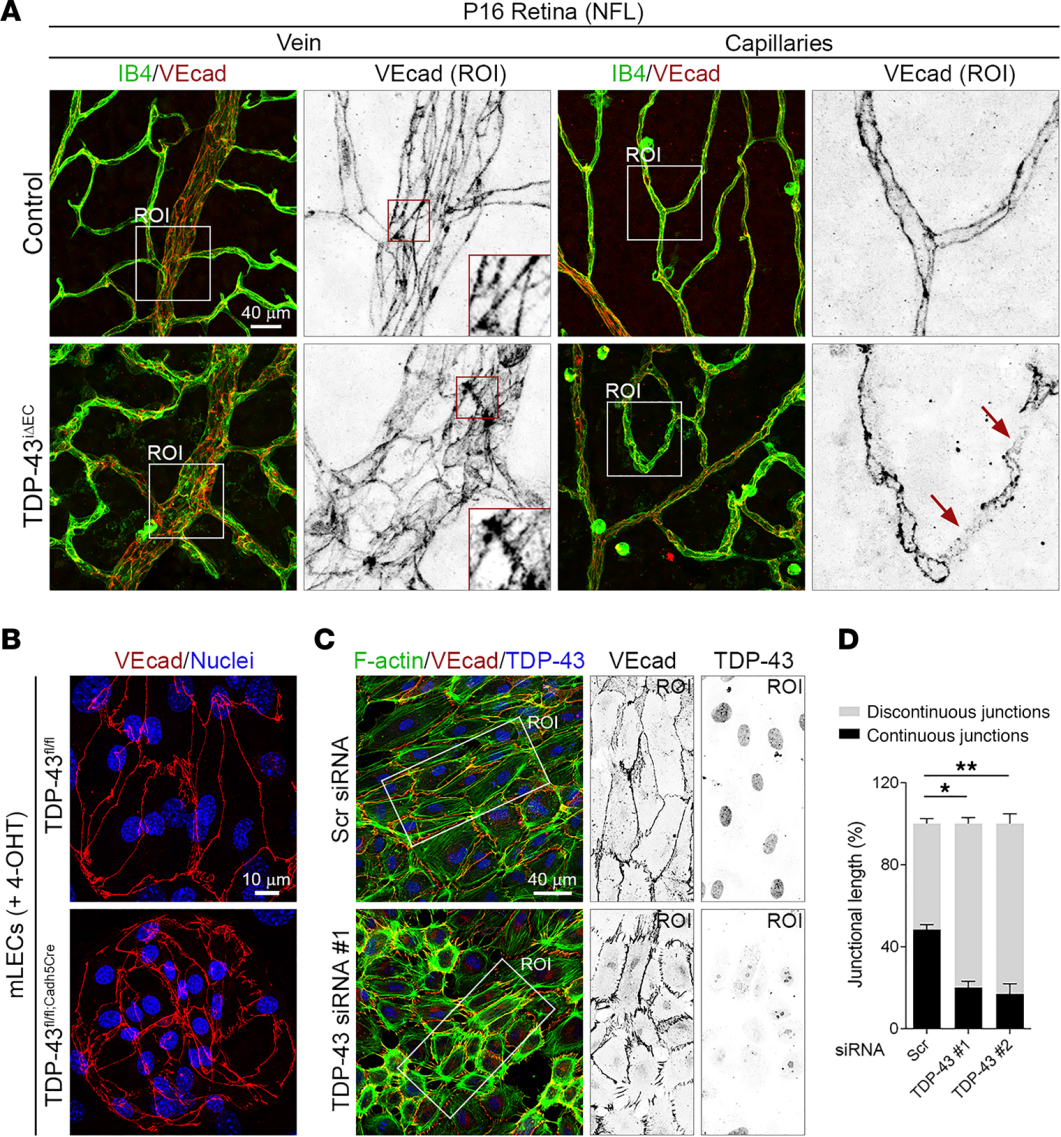
Loss of TDP-43 disrupts endothelial adherens junctions. (**A**) Confocal high-magnification images of P16 control and *TDP-43*^i*Δ*EC^ retinas stained for IB4 (green) and VE-cadherin (VEcad; red). White squares indicate the magnified ROIs. Arrows highlight vessel segments with diffuse punctuated VEcad stain. Scale bar: 40 μm. (**B**) Confocal high-magnification images of TDP-43^fl/fl^ and TDP-43^fl/fl;Cadh5Cre^ mLECS stained for VEcad (red) and Hoechst (nuclei, blue). Scale bar: 10 μm. (**C**) Confocal high-magnification images of HUVECs transfected with Scr or TDP-43 siRNAs stained for F-actin (green), VEcad (red), and TDP-43 (blue). White rectangles indicate the magnified ROIs. Scale bar: 40 μm. (**D**) Quantification of percentages of continuous (stable) and discontinuous (remodeling) adherens junctions in control and *TDP-43–*depleted HUVECs. Data are shown as mean ± SEM. *P < 0.05, **P < 0.01. One-way ANOVA.

VE-cadherin staining of P16 control and *TDP-43^iΔEC^* retinas revealed several morphological and junctional alterations in the vasculature of *TDP-43^iΔEC^* mice (Figure 4A). In vessels of control retinas, ECs were elongated and showed a spindle-like morphology with continuous, straight adherens junctions (AJs) running longitudinally along the vessel axis (Figure 4A). In contrast, ECs from *TDP-43^iΔEC^* vessels showed a less elongated round morphology, and their AJs appeared irregular with a zig-zagged distribution of VE-cadherin (Figure 4A). In addition, whereas ECs from control capillaries showed linear and thin AJs, ECs from *TDP-43^iΔEC^* capillaries showed tortuous junctions with diffuse and discontinuous VE-cadherin stain (Figure 4A). Shear stress regulates AJ morphology (37). To determine whether the altered AJ morphology observed in *TDP-43^iΔEC^* vessels was independent of shear stress signals, we analyzed AJ morphology in mouse and human EC cultures under static conditions. Similar to the situation in *TDP-43^iΔEC^* mice, VE-cadherin staining of control and TDP-43–depleted ECs showed altered AJ morphology in TDP-43–depleted ECs (Figure 4, B and C). In contrast to control cells, in which a high fraction of VE-cadherin stain was distributed in a linear, continuous pattern along cell-cell borders, junctional VE-cadherin signals in TDP-43–depleted ECs were distributed in discontinuous finger-like structures oriented perpendicular to cell-cell borders (Figure 4, B and C). Quantitative analysis showed decreased levels of linear AJs in TDP-43–depleted ECs compared with control ECs (Figure 4D). Linear AJs, also called stable AJs, are associated with thin actin filaments running parallel to cell membranes, whereas finger-like AJs are connected to thick stress fibers (37). F-actin staining of control and TDP-43–depleted ECs showed that knockdown of TDP-43 also induces changes in the actin cytoskeleton, which were characterized by the formation of a prominent cortical F-actin belt and an increase in short radial actin bundles oriented perpendicular to interrupted AJs (Figure 4C and Supplemental Figure 4A). Similar F-actin defects were observed in ECs of *TDP-43^iΔEC^* retinas (Supplemental Figure 4B). Together, these results indicate that TDP-43 regulate AJ stability independently of shear stress signals.

**Figure 5:**
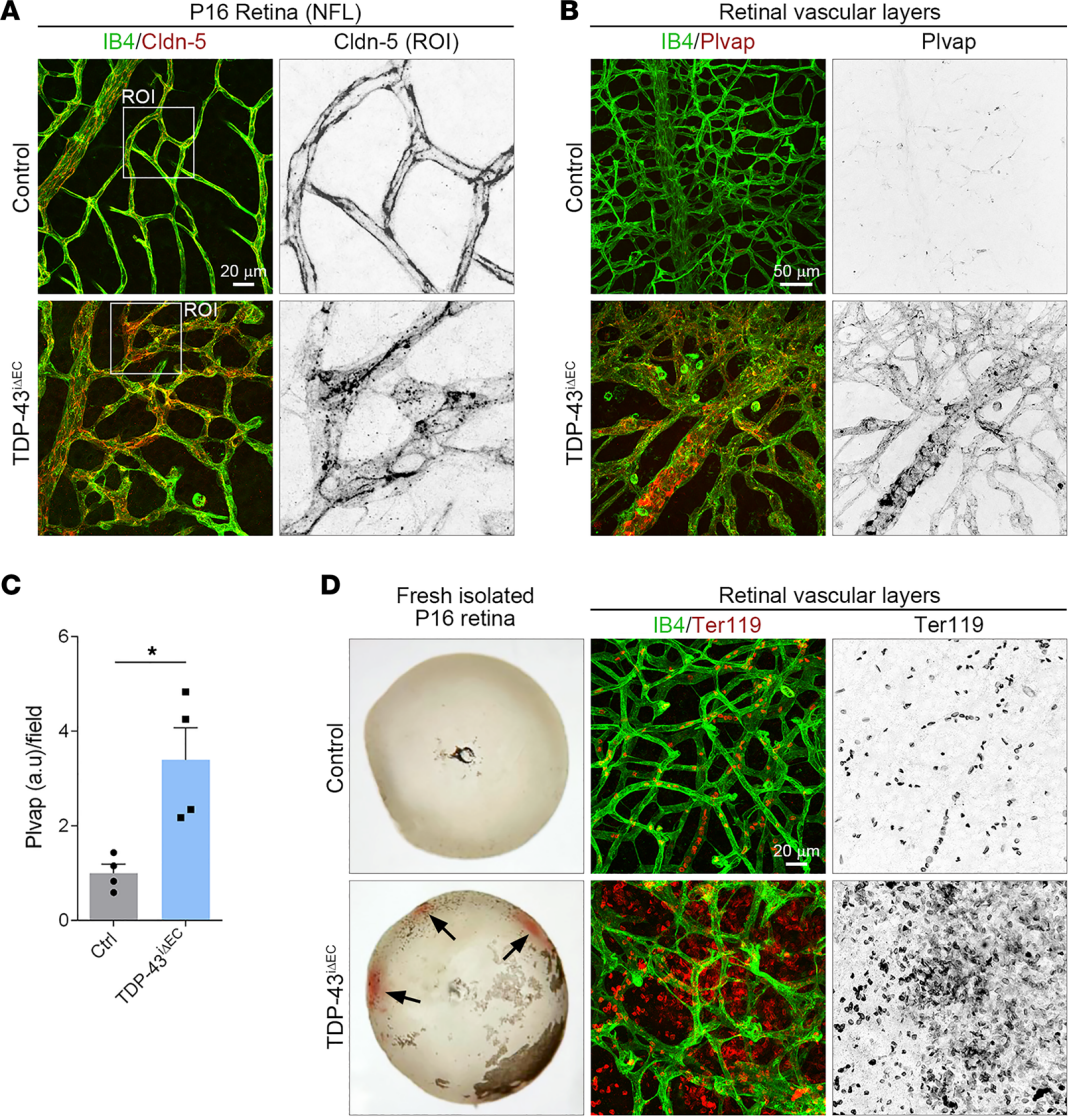
Endothelial TDP-43 is critical for BRB. (**A**) Confocal high-magnification images of P16 control and *TDP-43*^i*Δ*EC^ retinas stained for IB4 (green) and the endothelial tight junction marker Claudin-5 (Cldn-5, red). White squares indicate the magnified ROIs. Note the discontinuous and diffuse Cldn-5 staining in *TDP-43*^i*Δ*EC^ vessels. Scale bar: 20 μm. (**B**) Confocal high-magnification images of P16 control and *TDP-43*^i*Δ*EC^ retinas stained for IB4 (green) and the EC fenestration marker Plvap (red). Note the enhanced Plvap staining in *TDP-43*^i*Δ*EC^ vessels. Scale bar: 50 μm. (**C**) Quantification of Plvap fluorescence intensity per field in P16 control and *TDP-43*^i*Δ*EC^ retinas. Data are shown as mean ± SEM. *P < 0.05. Mann-Whitney *U* test. (**D**) Hemorrhages (arrows) in freshly isolated P16 retina of *TDP-43*^i*Δ*EC^ mice and confocal high-magnification images of P16 control and *TDP-43*^i*Δ*EC^ retinas stained for IB4 (green) and the RBC marker Ter119 (red). Note RBC leakage in *TDP-43*^i*Δ*EC^ vessels. Scale bar: 20 μm.

CNS ECs form TJs to establish the blood-CNS barrier (3). The distribution of claudin-5, a structural component of the endothelial TJs, was also altered in *TDP-43^iΔEC^* retinal vessels (Figure 5A). Whereas in control vessels, claudin-5 decorated cell-cell junctions, in *TDP-43^iΔEC^* vessels, claudin-5 was found in punctate cytoplasmic clusters, and its junctional distribution was diffuse and discontinuous (Figure 5A). Morphological defects in the cell-cell junctions of the *TDP-43^iΔEC^* vessels were accompanied by elevated staining signal of the EC fenestration component Plvap (Figure 5, B and C), a widely used marker of blood-CNS barrier disruption (11, 38). Consistent with the cell-cell junction defects, freshly isolated *TDP-43^iΔEC^* retinas showed extensive hemorrhages (Figure 5D). Immunostaining for the RBC marker Ter119 confirmed RBC extravasation in *TDP-43^iΔEC^* retinas but not in control retinas (Figure 5D). These findings indicated that deletion of endothelial TDP-43 impairs AJ stability and TJ morphology, compromises BRB function, and causes hemorrhages.

### Loss of TDP-43 in ECs leads to hemorrhages and blood vessel degeneration in the CNS.

**Figure 6:**
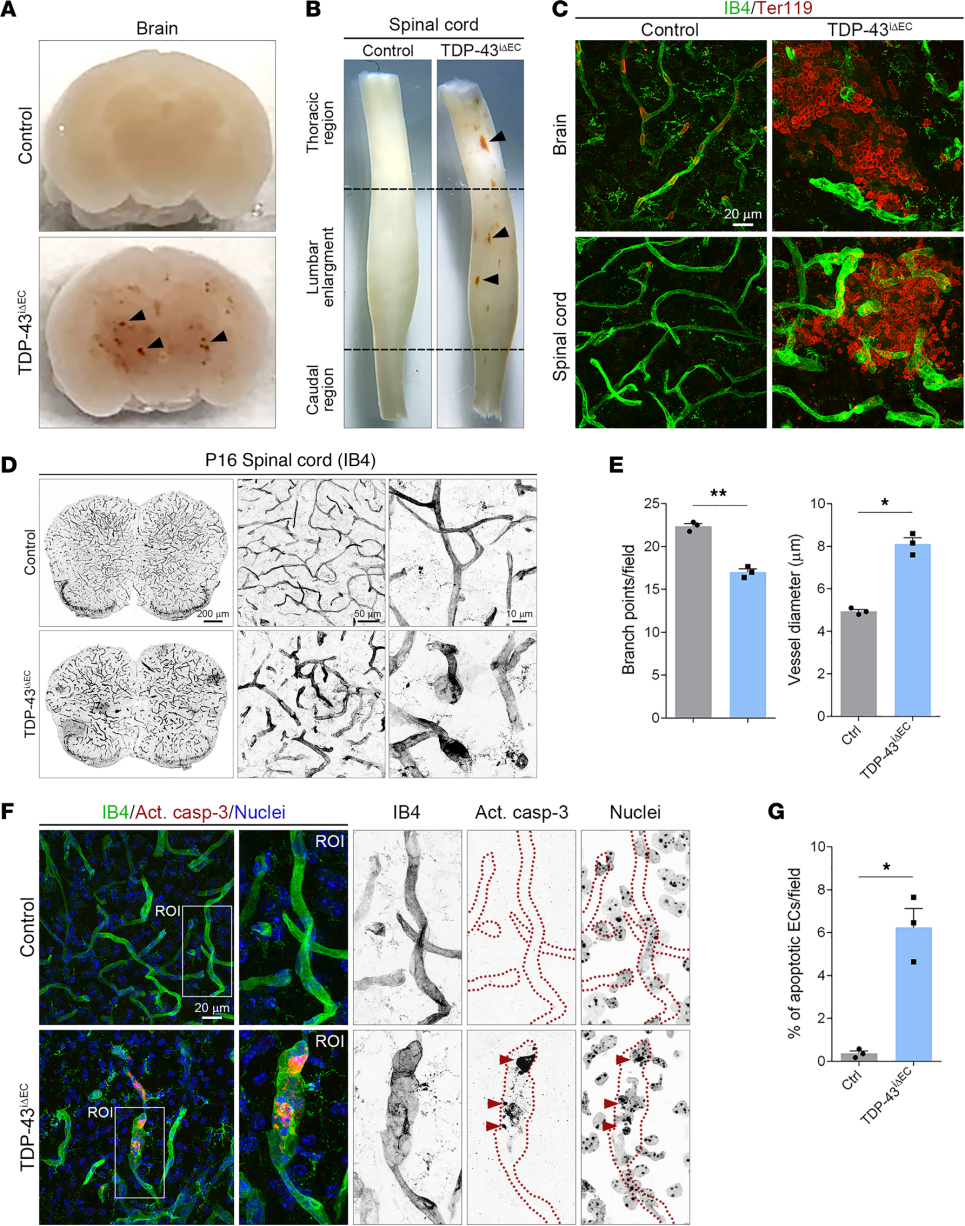
Hemorrhages and blood vessel degeneration in the CNS of *TDP-43^iΔEC^* mice. (**A** and **B**) Hemorrhages (arrowheads) in freshly isolated P16 brain (**A**) and spinal cord (**B**) of *TDP-43*^i*Δ*EC^ mice. (**C**) Confocal high-magnification images of P16 brain and spinal cord sections stained for IB4 (green) and Ter119 (red). Note the RBC leakage in *TDP-43*^i*Δ*EC^ vessels. Scale bar: 20 μm. (**D**) Low-magnification overview images and confocal high-magnification images of P16 control and *TDP-43*^i*Δ*EC^ spinal cord sections stained for IB4. Note the abnormal morphology of *TDP-43*^i*Δ*EC^ spinal cord vessels. Scale bars: 200 μm (left); 50 μm (center); and 10 μm (right). (**E**) Quantification of vascular parameters in P16 control and *TDP-43*^i*Δ*EC^ spinal cord sections. Data are shown as mean ± SEM. *P < 0.05, **P < 0.01. Mann-Whitney *U* test. (**F**) Confocal high-magnification images of P16 control and *TDP-43*^i*Δ*EC^ spinal cord sections stained for IB4 (green), the apoptosis marker active caspase-3 (Act. casp-3, red), and Hoechst (nuclei, blue). White rectangles indicate the magnified ROIs. Arrowheads indicate the active caspase-3^+^ ECs in *TDP-43*^i*Δ*EC^ mice. Dotted lines highlight the endothelial border of capillaries. Scale bars: 20 μm. (**G**) Quantification of the mean percentage of apoptotic ECs in P16 control and *TDP-43*^i*Δ*EC^ spinal cords. Data are shown as mean ± SEM. *P < 0.05. Mann-Whitney *U* test.

Vascular barrier defects in *TDP-43^iΔEC^* mice were not limited to the retina and focal hemorrhages, and accumulation of extravasated RBC also occurred in the brain and spinal cord of *TDP-43^iΔEC^* mice (Figure 6, A–C, and Supplemental Figure 5, A and B). Thus, to further characterize the role of TDP-43 in CNS ECs, we performed a quantitative and morphological analysis of the spinal cord vasculature of P16 control and *TDP-43^iΔEC^* mice. The analysis showed reduced vessel branching, increased vessel diameter, and irregular vessel shapes with balloon-like protrusions in *TDP-43^iΔEC^* mice compared with control mice (Figure 6, D and E). In mice, whereas retinal angiogenesis occurs exclusively at postnatal stages, angiogenesis in the spinal cord and brain occurs mainly during embryonic development (39). Therefore, we assessed whether the reduced vascularization of *TDP-43^iΔEC^* spinal cords was due to vessel degeneration. Immunostaining for anti–cleaved (active) caspase-3, a marker for apoptosis, on spinal cord sections from control and *TDP-43^iΔEC^* mice revealed elevated rate of apoptotic ECs in *TDP-43^iΔEC^* mice compared with control mice (Figure 6, F and G). Vascular malformations and degeneration were also observed in brain samples of P16 *TDP-43^iΔEC^* mice (Supplemental Figure 5, C and D). These results indicate that TDP-43 is required for vascular barrier integrity and EC survival in spinal cord and brain blood vessels.

### Reduced endothelial β-catenin signaling in TDP-43^iΔEC^ mice.

**Figure 7:**
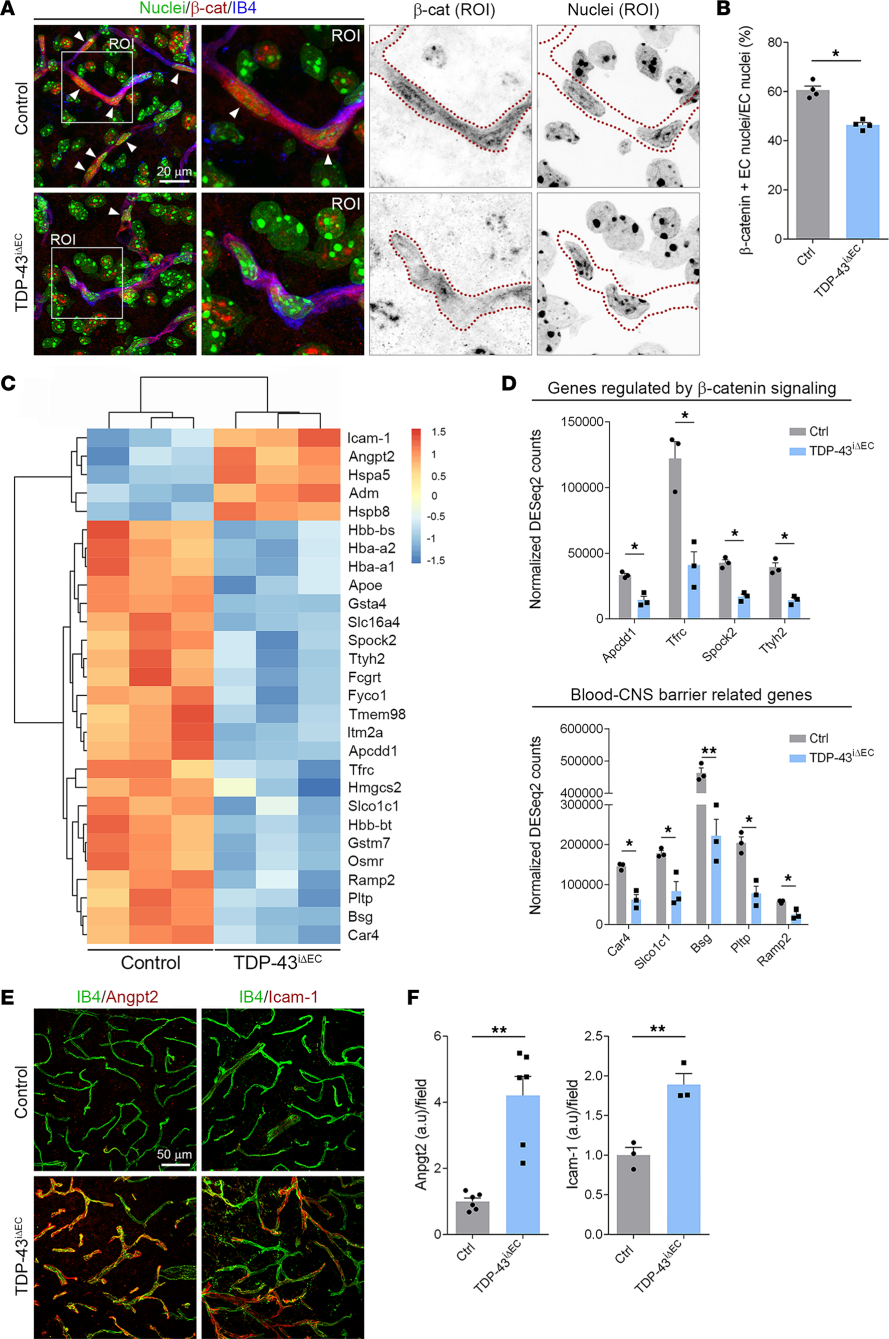
Reduced β-catenin signaling in *TDP-43^iΔEC^* ECs. (**A**) Confocal high-magnification images of P16 control and TDP-43^i*Δ*EC^ spinal cord sections stained for Hoechst (nuclei, green), β-catenin (β-cat, red), and IB4 (blue). White squares indicate the magnified ROIs. Arrowheads indicate the presence of nuclear β-catenin stain in ECs. Dotted lines highlight the endothelial border of capillaries. Scale bars: 20 μm. (**B**) Quantification of the relative percentage of ECs nuclei positive for β-catenin stain in P16 control and *TDP-43*^i*Δ*EC^ spinal cord. Data are shown as mean ± SEM. *P < 0.05. Mann-Whitney *U* test. (**C**) Heatmap representation of the differentially expressed genes (DEG) identified by transcriptomic analysis in ECs isolated from control and *TDP-43*^i*Δ*EC^ P16 spinal cord. (**D**) Detailed comparison of expression levels for DEGs regulated by β-catenin signaling and blood-CNS barrier in spinal cord ECs from control and *TDP-43*^i*Δ*EC^. Data are shown as mean ± SEM. *P < 0.05, **P < 0.01. Log_2_FC *P* value corrected with Benjamini-Hochberg. (**E**) Confocal high-magnification images of P16 control and *TDP-43*^i*Δ*EC^ spinal cord sections stained for IB4 (green), angiopoietin-2 (Angpt2, red), and Icam-1 (red). Scale bars: 50 μm. (**F**) Quantification of Angpt2 and Icam-1 fluorescence intensity per field in P16 control and *TDP-43*^i*Δ*EC^ spinal cord sections. Data are shown as mean ± SEM. **P < 0.01. Mann-Whitney *U* test.

Endothelial β-catenin signaling is crucial for vessel ingression into the CNS parenchyma and maintenance of blood-CNS barrier integrity (12). To address the possibility of defective endothelial β-catenin transcription activity in *TDP-43^iΔEC^* mice, we analyzed β-catenin localization in spinal cord ECs. β-Catenin staining of P16 control and *TDP-43^iΔEC^* spinal cord section revealed decreased nuclear β-catenin staining in ECs, but not in surrounding non-ECs, of *TDP-43^iΔEC^* mice compared with control mice (Figure 7A). Quantification analysis showed 20% reduction in the number of ECs with nuclear β-catenin staining in *TDP-43^iΔEC^* mice (Figure 7B). Next, to confirm defective β-catenin transcriptional activity in *TDP-43^iΔEC^* ECs, we used FACS to isolate ECs from spinal cords of P16 control and *TDP-43^iΔEC^* mice, and performed bulk RNA-Seq (Supplemental Figure 6A). Sequencing analysis showed significant enrichment of EC marker genes, including *Pecam1*, *Cldn5*, *Kdr*, *Flt1*, and *vWF*, confirming the endothelial identity of sorted cells (Supplemental Figure 6B). In addition, gene signatures assigned to other CNS cell populations, such as vascular mural cells, neurons, astrocytes, and microglial cells, appeared at relatively low counts, thus validating our specific sorting strategy for spinal cord ECs (Supplemental Figure 6B). As expected, differential expression analysis showed strong downregulation of the *Tardbp* gene in *TDP-43^iΔEC^* cells compared with control cells (Supplemental Figure 6C). The analysis identified 28 genes with altered expression in *TDP-43^iΔEC^* cells (5 upregulated and 23 downregulated) (Figure 7C). Among the genes that were downregulated in *TDP-43^iΔEC^* cells, 4 (*Apcdd1*, *Tfrc*, *Spock2*, and *Ttyh2*) encoded for proteins activated in response to β-catenin signaling, implying defective β-catenin signaling in these cells (40–42) (Figure 7D). Interestingly, 3 of these genes (*Apcdd1*, *Tfrc*, and *Spock2*) also meet the criteria for inclusion as “blood-CNS barrier genes,” in accordance with the established role of β-catenin signaling in BBB development. *TDP-43^iΔEC^* cells also showed reduced expression of other BBB-related genes (*Car4*, *Slco1c1*, *Bsg*, *Pltp*, and *Ramp2*) (40–44) (Figure 7C). Taken together, these findings establish a functional link between TDP-43 and β-catenin signaling in CNS ECs.

### TDP-43^iΔEC^ mice exhibit CNS inflammation.

**Figure 8:**
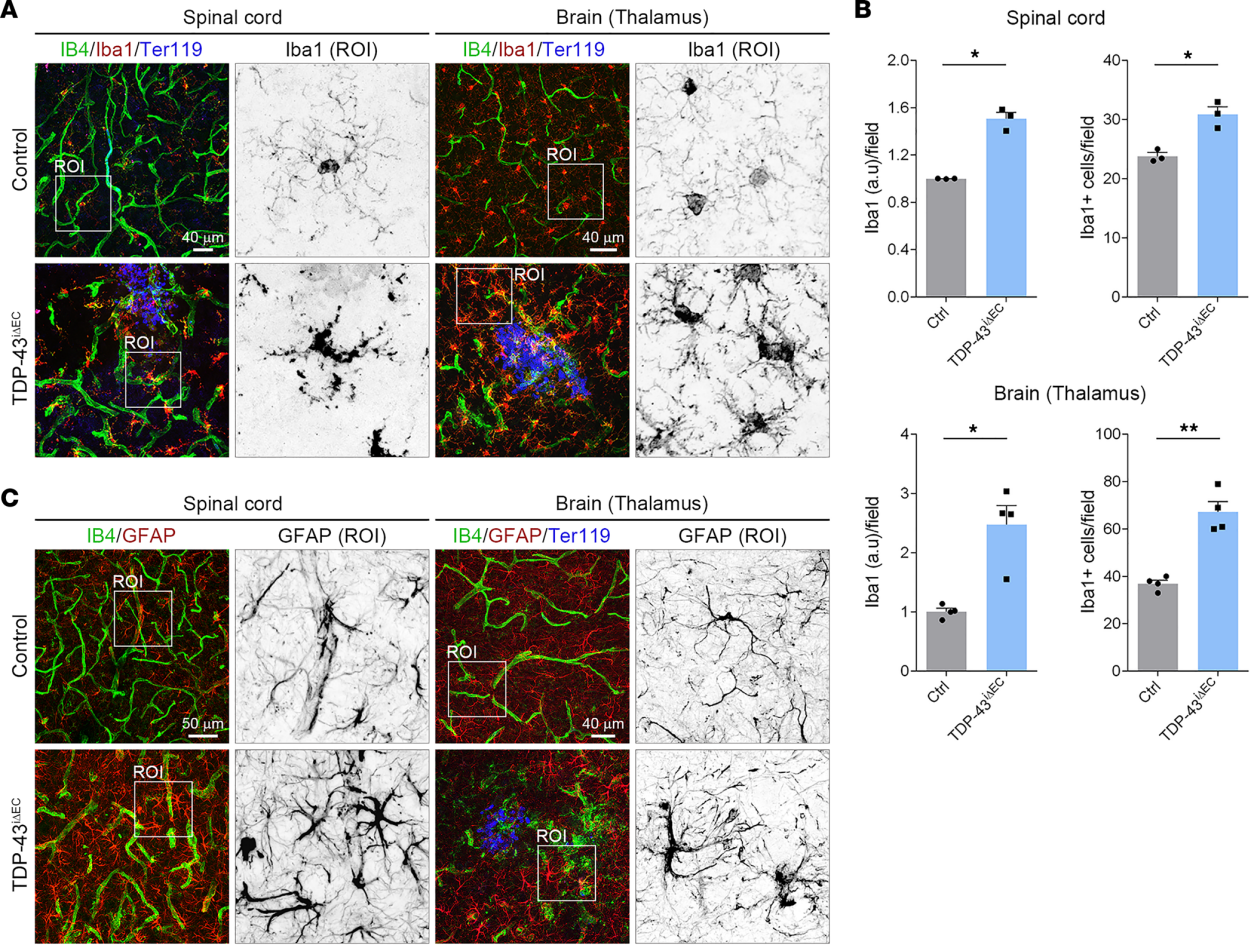
Loss of endothelial TDP-43 triggers inflammation in the CNS. (**A**) Confocal high-magnification images of P16 control and *TDP-43*^i*Δ*EC^ spinal cord and brain sections stained for IB4 (green), the microglial marker Iba1 (red), and Ter119 (blue). White squares indicate the magnified ROIs. Note the enhanced Iba1 staining in *TDP-43*^i*Δ*EC^ samples and the morphological differences between Iba1^+^ cells from control and *TDP-43*^i*Δ*EC^ mice. Scale bars: 40 μm. (**B**) Quantification of Iba1 fluorescence intensity per field and total Iba1^+^ cells per field in P16 control and *TDP-43*^i*Δ*EC^ spinal cord and brain as indicated. Data are shown as mean ± SEM. *P < 0.05, **P < 0.01. Mann-Whitney *U* test. (**C**) Confocal high-magnification images of P16 control and *TDP-43*^i*Δ*EC^ spinal cord and brain sections stained for IB4 (green), the astrocytic marker GFAP (red), and Ter119 (blue). White squares indicate the magnified ROIs. Note the presence of reactive astrocytes in *TDP-43*^i*Δ*EC^ spinal cord and brain. Scale bars: 50 μm (spinal cord) and 40 μm (brain).

Consistent with the vascular degeneration observed in *TDP-43^iΔEC^* mice, *TDP-43^iΔEC^* ECs also showed increased expression levels of the angiopoietin-2 (Angpt2), a vascular destabilizing factor, and intercellular adhesion molecule-1 (Icam-1), an inflammatory marker (Figure 7C). Immunostaining analysis confirmed increased Angpt2 and Icam-1 protein levels in *TDP-43^iΔEC^* spinal cord vessels (Figure 7, E and F). In addition, elevated Angpt2 and Icam-1 protein levels were also observed in brain and retinal blood vessels of *TDP-43^iΔEC^* mice (Supplemental Figure 7, A and B). Vascular dysfunction in the CNS triggers an inflammatory response involving activation of microglia and astrocytes, which respond to vascular injury with programs that include proliferation, morphological and genetic changes, and engulfment of extravasated blood cells and subcellular elements (3, 6, 45). Staining for Iba1, a marker of mature microglia (46), revealed morphological changes in microglia of *TDP-43^iΔEC^* mice (Figure 8A). In contrast to microglia of control mice, which showed a ramified morphology with long, thin branches and comparatively small cell bodies, typical of the surveying phenotype, microglia of *TDP-43^iΔEC^* mice showed a bushy morphology with short, swollen branches and enlarged cell bodies, characteristic of the activated phenotype (Figure 8A). These morphological changes were associated with an elevated Iba1 staining signal and an increased number of Iba1^+^ cells in *TDP-43^iΔEC^* mice compared with control mice (Figure 8B). Microglia activation was also observed in the retinas of *TDP-43^iΔEC^* mice (Supplemental Figure 7, C and D). After vessel rupture, activated microglia and monocyte-derived macrophages are rapidly recruited to the bleeding sites to eliminate extravasated RBCs by erythrophagocytosis (47). RBCs engulfed by IB4^+^ macrophages were frequently observed in *TDP-43^iΔEC^* mice (Supplemental Figure 7E). The most prominent morphological features of reactive astrocytes are cell body hypertrophy and increased glial fibrillary acidic protein (GFAP) expression (6). Immunostaining of GFAP revealed abundant astrocytes with an activated phenotype morphology in *TDP-43^iΔEC^* mice but not in control mice (Figure 8C). Together, these findings indicated that deletion of endothelial TDP-43 triggers inflammation in the CNS.

## Discussion

The coordination between the formation of a dense vascular network and the establishment of a functional blood-CNS barrier during development is essential for CNS homeostasis and function. Disruption of blood-CNS barriers elicit inflammatory and immune responses that can initiate multiple pathways of neurodegeneration (6). In the present study, we show that endothelial TDP-43, a neurodegeneration-associated protein, controls retinal vascularization, blood-CNS barrier integrity, and CNS vessel stability. Mechanistically, we show that TDP-43 is required for proper FN matrix assembly during vessel sprouting and β-catenin signaling in ECs.

The CNS is mainly vascularized through sprouting angiogenesis, a highly regulated process that involves coordinated EC specification, proliferation, and migration (12). At the onset of angiogenesis, ECs interact via integrin receptors with ECM proteins. These interactions promotes EC proliferation and migration during vessel sprouting and the stabilization of the nascent vasculature (9, 10). FN is a major component of the ECM and plays a critical role in blood vessel formation (48). It is involved in the binding and assembly of ECM proteins around the vessels, provides structural support for EC adhesion, and controls the availability of growth factors. During retinal sprouting angiogenesis, astrocytes and ECs secrete and assemble a fibrillar FN matrix that promotes vessel growth. The astrocyte-derived FN matrix promotes EC migration to avascular areas of the retina and limits excessive vessel sprouting, whereas the EC-derived FN matrix promotes sprouting and branching of the vasculature (33, 34). Here we show that postnatal EC-specific deletion of TDP-43 leads to retinal hypovascularization due to defects in vessel sprouting associated with reduced EC proliferation and migration. High-magnification imaging illustrated a highly disorganized FN matrix, with many irregular aggreates and few fibrils, around sprouting vessels in P7 *TDP-43^iΔEC^* retinas. In vitro, FN matrix defects in TDP-43–depleted ECs correlate with reduced FA formation and cell spreading. Taken together, these findings establish that TDP-43 controls sprouting angiogenesis in the retina, in part, by regulating endothelial FN matrix assembly and integrin-mediated cell-ECM adhesion. This conclusion is further supported by our recent publication showing that *FN1* is a direct RNA target of TDP-43 in EC and that deletion of TDP-43 in zebrafish embryos leads to sprouting defects during intersegmental vessel development associated to defective FN-integrin signaling (29).

Endothelial TDP-43 is also critical for vessel ingression into the deep retina and for cell-cell junction integrity. *TDP-43^iΔEC^* retinas show incomplete intraretinal capillary network formation, accumulation of globular EC clusters around the superficial vascular layer, disorganized cell-cell junctions, and intraocular hemorrhages. High-resolution imaging of EC-monolayers in vitro showed that TDP-43 is required for AJ stabilization and actin cytoskeleton arrangement. Endothelial β-catenin signaling is crucial for vessel ingression into the CNS parenchyma and for the establishment and maintenance of BBB integrity (12). Defective Wnt/β-catenin signaling has been linked to familial exudative vitreoretinopathy (49), a genetic disorder in which angiogenesis is impaired in the retina of young children (50), and to various CNS disorders involving BBB disruption, such as multiple sclerosis, stroke, and Alzheimer’s disease (6, 7). Dysregulation of Wnt/β-catenin signaling in mice leads to multiple sprouting angiogenesis defects in the CNS, including absence of deep retinal vasculature, formation of ball-shaped EC aggregates around the superficial retinal layer, retinal hemorrhages, and disruption of the BBB (51). The similarities between the retinal vascular phenotype of *TDP-43^iΔEC^* mice and retinal vascular defects caused by defective Wnt/β-catenin signaling argue for a functional link between TDP-43 and β-catenin signaling in ECs. Supporting this functional requirement of TDP-43 for proper β-catenin signaling, we show that loss of TDP-43 leads to reduced nuclear levels of β-catenin in CNS ECs, downregulation of β-catenin–regulated genes, and disruption of BBB and BSCB. In line with our findings, it has been recently shown that TDP-43 activates Wnt/β-catenin signaling in hepatocellular carcinoma cells during metastasis (52).

Disruption of CNS-vascular barriers results in the entry of blood plasma, cells, and microbial agents into the CNS parenchyma and is associated with activation of microglia and astrocytes, leading to inflammatory and immune responses that can initiate multiple neurodegeneration pathways (6). Indeed, CNS vasculature pathologies, including BBB disruption and EC degeneration, have recently emerged as early events in the pathogenesis of several neurodegenerative disorders, including Alzheimer’s disease, ALS, and LATE (6, 7, 22, 53). However, the exact cause and pathogenesis of many of these neurodegenerative disorders remains unknown, making it difficult to know whether BBB dysfunction in disease is a causative agent or a consequence of the disease (6, 7). ALS is a multifactorial neurodegenerative disorder characterized by progressive degeneration of upper and lower motor neurons, leading to muscle atrophy and death (54). In addition to neuronal degeneration and muscle atrophy, patients with ALS exhibited endothelial damage and impaired vascular barrier function (55–57). Histological analysis of brain and spinal cord tissue samples from patients with ALS revealed endothelial degeneration with reduced TJ (6). In a mouse model of ALS, TJ defects and BSCB disruption precede the onset of motor neuron degeneration and neurovascular inflammatory response (58). Moreover, decreased vessel perfusion results in selective motor neuron degeneration and muscle weakness, reminiscent of ALS (59). These findings suggest that endothelial damage, impaired CNS-vascular barrier function, and chronic vascular insufficiency might play a role in the onset and/or progression of ALS, preceding neuronal loss.

The etiology of ALS is mostly sporadic, and only 5%–10% of cases are genetically linked; in 98% of patients with ALS, motor neuron degeneration is accompanied by nuclear clearance and cytoplasmic aggregation of TDP-43 protein. Patients with sporadic ALS also show misregulation of FN, alterations in cell-ECM adhesion, and reduced vascular density in the spinal cord due to blood vessel regression (6, 60, 61). A recent report showed TDP-43 proteinopathy in vascular cells of spinal cords from patients with ALS, suggesting a loss of TDP-43 function in endothelium of these patients (21). Our results show that TDP-43 is essential for EC survival and that loss of endothelial TDP-43 leads to vascular degeneration, reduced vessel density in the spinal cord, and neuroinflammation. These vascular defects are associated with an upregulation in ECs of the vascular destabilizing factor Angpt2 and the inflammatory factor Icam-1. Consistent with the critical role of endothelial TDP-43 in blood vessel integrity and vascular function, its deletion in mice causes lethality within 2–3 weeks. Interestingly, loss of TDP-43 function in zebrafish results in severe vascular defects prior to the onset of neuronal degeneration (28). Together, these observations raise the question whether loss or malfunction of TDP-43 in the endothelium plays a role in the initiation and/or progression of ALS. Therefore, it would be of great interest to determine whether impaired function of TDP-43 in the endothelium of mature vessels are involved in ALS or other TDP-43–associated pathologies with increased vascular permeability and/or inflammation.

In summary, our study identifies endothelial TDP-43 as an important regulator of vascular growth and integrity, and paves the way for a new body of research on the role of TDP-43 in vascular cells.

## Methods

### Sex as a biological variable.

Sex was not considered a biological variable in this study, so mixed groups of male and female mice were used.

### Mouse lines, inducible genetic experiments, and ethical statement.

Mice were kept in the animal facilities of the University of Barcelona. *Cdh5(PAC)-Cre^ERT2^* mice (31) were bred with *Tardbp^fl^* mice (The Jackson Laboratory, stock no. 017591) carrying *loxP* sites flanking exon 3 of the *Tdp-43* gene (*Tardbp*) to obtain *Cdh5(PAC)-Cre^ERT2^;Tardbp^fl^* mice on a C57BL/6 genetic background. Postnatal gene deletion in newborn mice was induced by i.p. injection of 50 μL of tamoxifen solution (MilliporeSigma, T5648, 1 mg/mL) generated by diluting a 10 mg/mL tamoxifen stock solution in 1:4 ethanol-peanut oil (MilliporeSigma, P2144) with additional peanut oil once daily at P1, P2, and P3 or P5, P6, and P7. Gene deletion in adult (8-week-old) mice was induced by i.p. injection of 1 mg tamoxifen once every 24 hours for a total of 3 consecutive days. Mice of both sexes were randomly assigned in every experimental group.

### IHC of whole-mount retina.

All immunostainings of the retina were carried out with littermates processed simultaneously under the same conditions, as described previously (62). Briefly, whole eyes were fixed in 4% paraformaldehyde (PFA) for 2 hours at 4°C and were washed with phosphate-buffered saline (PBS) before retinas were dissected. After blocking/permeabilization overnight at 4°C in blocking solution (1% BSA [MilliporeSigma, A4378] with 0.3% Triton X-100 [MilliporeSigma, T8787] in PBS), retinas were incubated overnight at 4°C with primary antibodies (Supplemental Table 1) diluted in blocking solution. The next day, the primary antibody solution was removed and retina samples were washed 5 times for at least 20 minutes with PBS. Next, retinas were incubated with fluorescence-coupled secondary antibodies (Supplemental Table 1) overnight at 4°C. After incubation, retinas were washed 5 times for at least 20 minutes with PBS. Finally, samples were partially cut in 4 quadrants mounted on a microscope slide using ProLong Gold (Invitrogen, P36934) mounting medium.

### Immunofluorescence staining of brain, spinal cord, and liver cryosections.

P16 mice were anesthetized with an i.p. injection of a ketamine (70 mg/kg; Pfizer) and xylacine (20 mg/kg; Calier) mixture and perfused intracardially with PBS. Brains, spinal cords, and livers were dissected and fixed in 4% PFA solution for 1 day at 4°C. The next day, samples were dehydrated in a 30% sucrose solution in PBS for 3 days, frozen in cryoembedding solution (Tissue-Tec O.C.T., Sakura-Finetek, 4583) and stored at –80°C. Frozen liver, brain, and spinal cord blocks were cut into 40 μm coronal sections and preserved as free-floating sections with cryoprotectant (ethylenglycol [MilliporeSigma, 324558], glycerol [MilliporeSigma, G5516], and 0.1M PBS in a 1:1:2 solution at pH 7.4). Floating sections stored in cryopreservative solution were extensively washed with PBS, immersed for 1 hour at room temperature (RT) in blocking-solution (PBS containing 5% BSA and 0.3% Triton X-100), and incubated overnight at 4°C with primary antibodies (Supplemental Table 1). After extensive washing with PBS, sections were incubated for 2 hours at RT with fluorescence-coupled secondary antibodies (Supplemental Table 1). After incubation, samples were washed 5 times for at least 20 minutes with PBS. Finally, samples were mounted in ProLong Gold medium (Thermo Fisher Scientific, P36934).

### Image acquisition and processing.

Stained and flat-mounted retinas and stained brain and spinal cord sections were analyzed at high resolution with the Zeiss LSM 880 laser scanning confocal microscope. A Carl Zeiss AxioImager M2 with Apotome module was used for lower resolution. Zen Black (Zeiss), IMARIS, ImageJ (NIH), and Photoshop CS (Adobe) software were used for image acquisition and processing. All images shown are representative of at least 6 different images of at least 3 mice per group with identical laser excitation and microscope detection settings.

### Quantitative analysis of retinal and spinal cord vasculature.

Quantification of vascular parameters in retinal samples was performed with littermate samples processed simultaneously under the same conditions, as described previously (63). Briefly, vascular outgrowth in P7 retinas was measured by defining a straight line from the angiogenic front to the center of the retina for each retina quadrant. Four quadrants from at least 14 retinas per group were used for quantification. The number of ECs, number of endothelial sprouts, and vessel diameter were calculated with ImageJ software. The number of ECs and the area covered by EC were calculated from 24 fields (sized 566 × 566 μm) from 8 retina samples per group. The number of endothelial sprouts at the angiogenic front quantification was calculated from 40 fields (sized 212 × 212 μm) from 10 retina samples per group. The number of vertical endothelial sprouts and the vessels diameter were calculated from 30 fields (sized 566 × 566 μm) from 6 retina samples per group. The number of branchpoints was calculated with AngioTool 0.6 software from at least 36 fields (size 414 × 414 μm) from 7 retinas per group. The number of branchpoints in spinal cord samples was calculated with AngioTool 0.6 software from 15 fields (sized 340 × 340 μm) from 3 mice per group.

### Cell culture and small interfering RNA transfection.

HUVECs purchased from PromoCell (catalog C-12203) were maintained in Endothelial Cell Growth (ECG) Medium (PromoCell, C-22010). HUVECs at passages 3–8 were used for the experiments. HUVECs seeded on 0.2% gelatin-coated (MilliporeSigma, G1393) dishes the day before transfection were transfected siRNA (50 pmols/mL) for human TDP-43 (MilliporeSigma, NM_007375; Hs01_00037054 and Hs01_00037055) and scramble control siRNA (Sigma-Aldrich, SIC001) using Lipofectamine RNAiMax (Invitrogen, 13778-150) according to the manufacturer’s protocol for 24 hours and changed to ECG medium. All experiments were done 72 hours after transfection and with the 2 TDP-43 siRNAs.

### Isolation and treatment of mLECs.

Mouse lungs were digested with Collagenase II (1 mg/mL, Thermo Fisher Scientific, 17101-015) in HBSS (Thermo Fisher Scientific, 14025-092) for 1 hour at 37°C, followed by positive selection with anti–mouse CD31 (BD Pharmingen, 553370) antibody coated with magnetic dynabeads sheep anti–rat IgG (Thermo Fisher Scientific, 11035). Cells were seeded on a 0.5% gelatin-coated 12-well plate in DMEM/F12 (Thermo Fisher Scientific, 21041025) supplemented with 20% FBS (Biological Industries, 04-007-1A) and EC growth supplement/heparin (PromoCell, C-30140). Afterward, the cells were reselected with CD31 antibody–coated magnetic beads. To induce gene deletion, 4-OHT (2 μM, MilliporeSigma, H7904) was added to the cultured medium at day 4 for 24 hours. All experiments were done 72 hours after 4-OHT treatment.

### Western blot analysis.

Cells were lysed in Laemmli sample buffer —Tris-HCl (pH 6.8), SDS 2%, glycerol 10% (MilliporeSigma, G5516) — with pepstatin A (1 μg/mL), leupeptin (1 μg/mL), aprotinin (1 μg/mL), benzamidine (1 mM), phenylmethylsulfonyl fluoride (PMSF, 1 mM), and Na_3_VO_4_ (1 mM). Protein concentration was measured with the Micro BCA Protein Assay Reagent kit (Thermo Fisher Scientific, 23235). Samples were boiled at 95°C for 5–10 minutes to denature the proteins. Lysates were resolved by SDS-PAGE gels. Proteins were then electrophoretically transferred from the gels to Immobilon-P membranes (MilliporeSigma, IPVH00010). Blots were blocked with 5% milk powder in Tris-buffered saline (TBS) with Tween-20 (TBST) for 1 hour at RT. Blots were incubated overnight at 4°C with primary antibodies (Supplemental Table 1) in 5% milk powder in TBST. The secondary antibodies linked to horseradish peroxidase (HRP) (Supplemental Table 1) were incubated with the membrane for 1 hour and 30 minutes at RT. As a final step before imaging, blots were washed with TBS. HRP signals were visualized by Amersham enhanced chemiluminescence (ECL) detection reagents (Cytiva, RPN2236) and visualized with Amersham Imager 680. Intensities of bands were quantified with Multi Gauge V3.0 software (Fujifilm Corp.).

### Immunofluorescence analysis of HUVECs and mLECs.

HUVECs and mECs were cultured on coverslips coated with gelatin 0.2% or 0.5% in PBS, respectively. Cells were fixed for 10 minutes at RT with 4% PFA in PBS. Fixed cells were permeabilized for 20 minutes at RT with 0.1% Triton X-100 in PBS and blocked for 2 hours at RT with blocking solution (PBS containing 3% BSA and 0.1% Triton X-100). Primary antibodies (Supplemental Table 1) were diluted in blocking solution and incubated overnight at 4°C. The next day, cells were washed 3 times with 0.1% Triton X-100 in PBS and incubated for 1 hour at RT with fluorescence-coupled secondary antibodies and phalloidin (Supplemental Table 1). Next, cells were incubated with Hoechst 3342 (Invitrogen, H3570) for 10 minutes at RT. After washing 3 times with 0.1% Tween-20 in PBS, coverslips were mounted in ProLong Gold solution.

Stained cells were analyzed at high resolution with the Zeiss LSM 880 laser scanning confocal microscope. Zen Black (Zeiss), IMARIS, ImageJ, and Photoshop CS (Adobe) software were used for image acquisition and processing. Cell-cell junctional length was quantified as previously described (63) using the ImageJ software using 5 fields (magnification, ×25) per sample from 4 independent experiments.

### EC sorting and RNA isolation.

To isolate ECs from P16 mice spinal cord, we followed the previously published protocol (64). Briefly, spinal cord tissue was dissected, minced, and digested with collagenase (1 mg/mL) for 30 minutes at 37°C with rotation, triturated in 2% FBS in 1× HBSS, and centrifuged (at 300 g for 5 minutes at 4°C) through 22% Percoll (MilliporeSigma, P1644) to remove debris. The samples were blocked with HBSS/BSA/glucose buffer and incubated for 20 minutes with fluorescently conjugated antibodies at 4°C. After washing, samples were resuspended in HBSS buffer with DAPI to exclude dead cells. Cells were sorted using FACS Aria III (BD Biosciences), and data were analyzed using FACSDiva software (BD Biosciences). All FACS gates were set using unlabeled cells as well as single-color and isotype controls from mice spinal cord samples. RNA was isolated from sorted ECs using RNeasy Micro Kit (Qiagen), following the manufacturer’s protocol.

### Low-input RNA-Seq.

RNA-Seq libraries were prepared following the SMARTseq2 protocol (65) with some modifications. Briefly, RNA was quantified using the Qubit RNA HS Assay Kit (Thermo Fisher Scientific), and the RNA integrity was estimated with Agilent RNA 6000 Pico Kit. Reverse transcription of the total RNA input material of 1.8 μL (0.2–3.1 ng, in function of the sample availability) was performed using SuperScrpit II (Invitrogen) in a presence of oligo-dT30VN (1 μM; 5′-AAGCAGTGGTATCAACGCAGAGTACT30VN-3′), template-switching oligonucleotides (1 μM) and betaine (1M). The cDNA was amplified using the KAPA Hifi Hotstart ReadyMix (Roche) with 100 nM IS PCR primer (5′-AAGCAGTGGTATCAACGCAGAGT-3′) by 15 or 20 cycles of single-primer cDNA amplification. Following purification with Agencourt Ampure XP beads (1:1 ratio; Beckmann Coulter), product size distribution and quantity were assessed on a Bioanalyzer High Sensitivity DNA Kit (Agilent). The amplified cDNA (200 ng) was fragmented for 10 minutes at 55°C using Nextera XT (Illumina) and amplified for 12 cycles with indexed Nextera PCR primers. The library was purified twice using Agencourt Ampure XP beads (0.8:1 ratio) and quantified on a Bioanalyzer using a High Sensitivity DNA Kit.

The libraries were sequenced on NovaSeq 6000 (Illumina) in paired-end sequencing method with a read length of 2 × 51 bp according to the manufacturer’s protocol for dual indexing. Image analysis, base calling, and quality scoring of the run are processed using the manufacturer’s software Real Time Analysis (RTA 3.4.4), followed by generation of FASTQ sequence files.

### RNA-Seq data processing and analysis.

RNA-Seq reads were mapped against the Mus musculus reference genome (GRCm39) with STAR/2.7.8a (66) using ENCODE parametres. Annotated genes (gencode.M30) were quantified with RSEM/1.3.0 (67) using default options. Differential expression analysis was performed with limma (68) using the voom (69) transformation. Functional enrichment of the differentially expressed genes (FDR < 5%) was done with gProfiler (70). The heatmap was performed using the pheatmap R Package and the multidimensional scaling plot using the plotMDS function of limma.

### Statistics.

Statistical analysis was performed using Mann-Whitney *U* test, 1-way ANOVA, or log-rank (Mantel-Cox) test, as indicated in the figure legends.

### Study approval.

All experiments with mice were performed in accordance to Spanish and German guidelines and regulations, and the protocols were approved by the Committee on the Ethics of Animal Experiments of the University of Barcelona and the government of Upper Bavaria (157_21 CEEA-UB and ROB-55. 2Vet2532.Vet_02-15-172).

### Data availability.

The RNA-Seq data set generated in this work has been deposited into the Gene Expression Omnibus (GEO) database (accession no. GSE253868). Values for all data points in graphs are reported in the Supporting Data Values file. Any additional information required to reanalyze the data reported in this paper is available from the corresponding author upon request.

## Author contributions

VA, PV, MS, OMME, BS, and EM designed experiments; VA, YO, MRP, and HB performed experiments; VA, MA, AEC, AM, and EM performed bioinformatic analysis; and VA and EM analyzed data and wrote the paper.

## Supplementary Material

Supplemental data

Unedited blot and gel images

Supporting data values

## Version 1 02/01/2024

In-Press Preview

## Version 2 03/08/2024

Electronic publication

## References

[1] Storkebaum E (2011). Cerebrovascular disorders: molecular insights and therapeutic opportunities. Nat Neurosci.

[2] Tata M (2015). Vascularisation of the central nervous system. Mech Dev.

[3] Zhao Z (2015). Establishment and dysfunction of the blood-brain barrier. Cell.

[4] Jun LY (2021). Blood-spinal cord barrier in spinal cord injury: a review. J Neurotrauma.

[5] O‘Leary F, Campbell M (2023). The blood-retina barrier in health and disease. FEBS J.

[6] Sweeney MD (2018). Blood-brain barrier breakdown in Alzheimer disease and other neurodegenerative disorders. Nat Rev Neurol.

[7] Sweeney MD (2018). The role of brain vasculature in neurodegenerative disorders. Nat Neurosci.

[8] Schaeffer S, Iadecola C (2021). Revisiting the neurovascular unit. Nat Neurosci.

[9] Hynes RO (2007). Cell-matrix adhesion in vascular development. J Thromb Haemost.

[10] Aman J, Margadant C (2023). Integrin-dependent cell-matrix adhesion in endothelial health and disease. Circ Res.

[11] Park H (2019). Integrin-linked kinase controls retinal angiogenesis and is linked to Wnt signaling and exudative vitreoretinopathy. Nat Commun.

[12] Wälchli T (2023). Shaping the brain vasculature in development and disease in the single-cell era. Nat Rev Neurosci.

[13] Ratti A, Buratti E (2016). Physiological functions and pathobiology of TDP-43 and FUS/TLS proteins. J Neurochem.

[14] Ling JP (2015). TDP-43 repression of nonconserved cryptic exons is compromised in ALS-FTD. Science.

[15] Neumann M (2006). Ubiquitinated TDP-43 in frontotemporal lobar degeneration and amyotrophic lateral sclerosis. Science.

[16] Gao J Pathomechanisms of TDP-43 in neurodegeneration. J Neurochem.

[17] Nag S, Schneider JA (2023). Limbic-predominant age-related TDP43 encephalopathy (LATE) neuropathological change in neurodegenerative diseases. Nat Rev Neurol.

[18] Sreedharan J (2008). TDP-43 mutations in familial and sporadic amyotrophic lateral sclerosis. Science.

[19] Ince PG (2011). Molecular pathology and genetic advances in amyotrophic lateral sclerosis: an emerging molecular pathway and the significance of glial pathology. Acta Neuropathol.

[20] Kapeli K (2017). Genetic mutations in RNA-binding proteins and their roles in ALS. Hum Genet.

[21] Ferrer I (2021). TDP-43 vasculopathy in the spinal cord in sporadic amyotrophic lateral sclerosis (sALS) and frontal cortex in sALS/FTLD-TDP. J Neuropathol Exp Neurol.

[22] Blevins BL (2021). Brain arteriolosclerosis. Acta Neuropathol.

[23] Polymenidou M (2011). Long pre-mRNA depletion and RNA missplicing contribute to neuronal vulnerability from loss of TDP-43. Nat Neurosci.

[24] Tollervey JR (2011). Characterizing the RNA targets and position-dependent splicing regulation by TDP-43. Nat Neurosci.

[25] Kraemer BC (2010). Loss of murine TDP-43 disrupts motor function and plays an essential role in embryogenesis. Acta Neuropathol.

[26] Sephton CF (2010). TDP-43 is a developmentally regulated protein essential for early embryonic development. J Biol Chem.

[27] Wu LS (2010). TDP-43, a neuro-pathosignature factor, is essential for early mouse embryogenesis. Genesis.

[28] Schmid B (2013). Loss of ALS-associated TDP-43 in zebrafish causes muscle degeneration, vascular dysfunction, and reduced motor neuron axon outgrowth. Proc Natl Acad Sci U S A.

[29] Hipke K (2023). Loss of TDP-43 causes ectopic endothelial sprouting and migration defects through increased fibronectin, vcam-1 and integrin α4/β1. Front Cell Dev Biol.

[30] Chiang PM (2010). Deletion of TDP-43 down-regulates Tbc1d1, a gene linked to obesity, and alters body fat metabolism. Proc Natl Acad Sci U S A.

[31] Wang Y (2010). Ephrin-B2 controls VEGF-induced angiogenesis and lymphangiogenesis. Nature.

[32] Potente M (2011). Basic and therapeutic aspects of angiogenesis. Cell.

[33] Turner CJ (2017). Endothelium-derived fibronectin regulates neonatal vascular morphogenesis in an autocrine fashion. Angiogenesis.

[34] Stenzel D (2011). Integrin-dependent and -independent functions of astrocytic fibronectin in retinal angiogenesis. Development.

[35] Stahl A (2010). The mouse retina as an angiogenesis model. Invest Ophthalmol Vis Sci.

[36] Mancuso MR (2006). Rapid vascular regrowth in tumors after reversal of VEGF inhibition. J Clin Invest.

[37] Huveneers S, de Rooij J (2013). Mechanosensitive systems at the cadherin-F-actin interface. J Cell Sci.

[38] Shue EH (2008). Plasmalemmal vesicle associated protein-1 (PV-1) is a marker of blood-brain barrier disruption in rodent models. BMC Neurosci.

[39] Biswas S (2020). Neuronal and glial regulation of CNS angiogenesis and barriergenesis. Development.

[40] Sabbagh MF, Nathans J (2020). A genome-wide view of the de-differentiation of central nervous system endothelial cells in culture. Elife.

[41] Jensen LD (2019). Disruption of the extracellular matrix progressively impairs central nervous system vascular maturation downstream of β-catenin signaling. Arterioscler Thromb Vasc Biol.

[42] Röhrs S (2009). Chronological expression of Wnt target genes Ccnd1, Myc, Cdkn1a, Tfrc, Plf1 and Ramp3. Cell Biol Int.

[43] Zhou T (2014). Phospholipid transfer protein (PLTP) deficiency impaired blood-brain barrier integrity by increasing cerebrovascular oxidative stress. Biochem Biophys Res Commun.

[44] Koyama T (2013). Vascular endothelial adrenomedullin-RAMP2 system is essential for vascular integrity and organ homeostasis. Circulation.

[45] Lan X (2017). Modulators of microglial activation and polarization after intracerebral haemorrhage. Nat Rev Neurol.

[46] Sasaki Y (2001). Iba1 is an actin-cross-linking protein in macrophages/microglia. Biochem Biophys Res Commun.

[47] Zhao X (2009). Hematoma resolution as a therapeutic target: the role of microglia/macrophages. Stroke.

[48] Astrof S, Hynes RO (2009). Fibronectins in vascular morphogenesis. Angiogenesis.

[49] Ye X (2010). The Norrin/Frizzled4 signaling pathway in retinal vascular development and disease. Trends Mol Med.

[50] Gilmour DF (2015). Familial exudative vitreoretinopathy and related retinopathies. Eye (Lond).

[51] Ye X (2009). Norrin, frizzled-4, and Lrp5 signaling in endothelial cells controls a genetic program for retinal vascularization. Cell.

[52] Guo F (2020). TDP-43 induces EMT and promotes hepatocellular carcinoma metastasis via activating Wnt/β-catenin signaling pathway. Am J Cancer Res.

[53] Ahmad A (2020). The role of neurovascular system in neurodegenerative diseases. Mol Neurobiol.

[54] Grad LI (2017). Clinical spectrum of amyotrophic lateral sclerosis (ALS). Cold Spring Harb Perspect Med.

[55] Garbuzova-Davis S (2011). Amyotrophic lateral sclerosis: a neurovascular disease. Brain Res.

[56] Garbuzova-Davis S (2012). Impaired blood-brain/spinal cord barrier in ALS patients. Brain Res.

[57] Winkler EA (2013). Blood-spinal cord barrier breakdown and pericyte reductions in amyotrophic lateral sclerosis. Acta Neuropathol.

[58] Zhong Z (2008). ALS-causing SOD1 mutants generate vascular changes prior to motor neuron degeneration. Nat Neurosci.

[59] Oosthuyse B (2001). Deletion of the hypoxia-response element in the vascular endothelial growth factor promoter causes motor neuron degeneration. Nat Genet.

[60] Lewandowski SA (2016). Presymptomatic activation of the PDGF-CC pathway accelerates onset of ALS neurodegeneration. Acta Neuropathol.

[61] Rabin SJ (2010). Sporadic ALS has compartment-specific aberrant exon splicing and altered cell-matrix adhesion biology. Hum Mol Genet.

[62] Pitulescu ME (2010). Inducible gene targeting in the neonatal vasculature and analysis of retinal angiogenesis in mice. Nat Protoc.

[63] Fraccaroli A (2015). Endothelial alpha-parvin controls integrity of developing vasculature and is required for maintenance of cell-cell junctions. Circ Res.

[64] Crouch EE, Doetsch F (2018). FACS isolation of endothelial cells and pericytes from mouse brain microregions. Nat Protoc.

[65] Picelli S (2014). Full-length RNA-Seq from single cells using Smart-seq2. Nat Protoc.

[66] Dobin A (2013). STAR: ultrafast universal RNA-Seq aligner. Bioinformatics.

[67] Li B, Dewey CN (2011). RSEM: accurate transcript quantification from RNA-Seq data with or without a reference genome. BMC Bioinformatics.

[68] Ritchie ME (2015). Limma powers differential expression analyses for RNA-sequencing and microarray studies. Nucleic Acids Res.

[69] Law CW (2014). Voom: precision weights unlock linear model analysis tools for RNA-Seq read counts. Genome Biol.

[70] Raudvere U (2019). g:Profiler: a web server for functional enrichment analysis and conversions of gene lists (2019 update). Nucleic Acids Res.

