# Supplementary material for: Endothelial TDP-43 controls sprouting angiogenesis and vascular barrier integrity, and its deletion triggers neuroinflammation
Source: JCI Insight. 2024 Feb 1;9(5):e177819. doi: 10.1172/jci.insight.177819 (PMC11143933; doi:10.1172/jci.insight.177819)
Supplement: Supplemental data [file jciinsight-9-177819-s068.pdf]

Supplemental Materials

Endothelial TDP-43 controls sprouting angiogenesis and vascular barrier integrity, and its deletion triggers neuroinflammation

Victor Arribas *et al.*

*Corresponding author. Email: emontanez@ub.edu

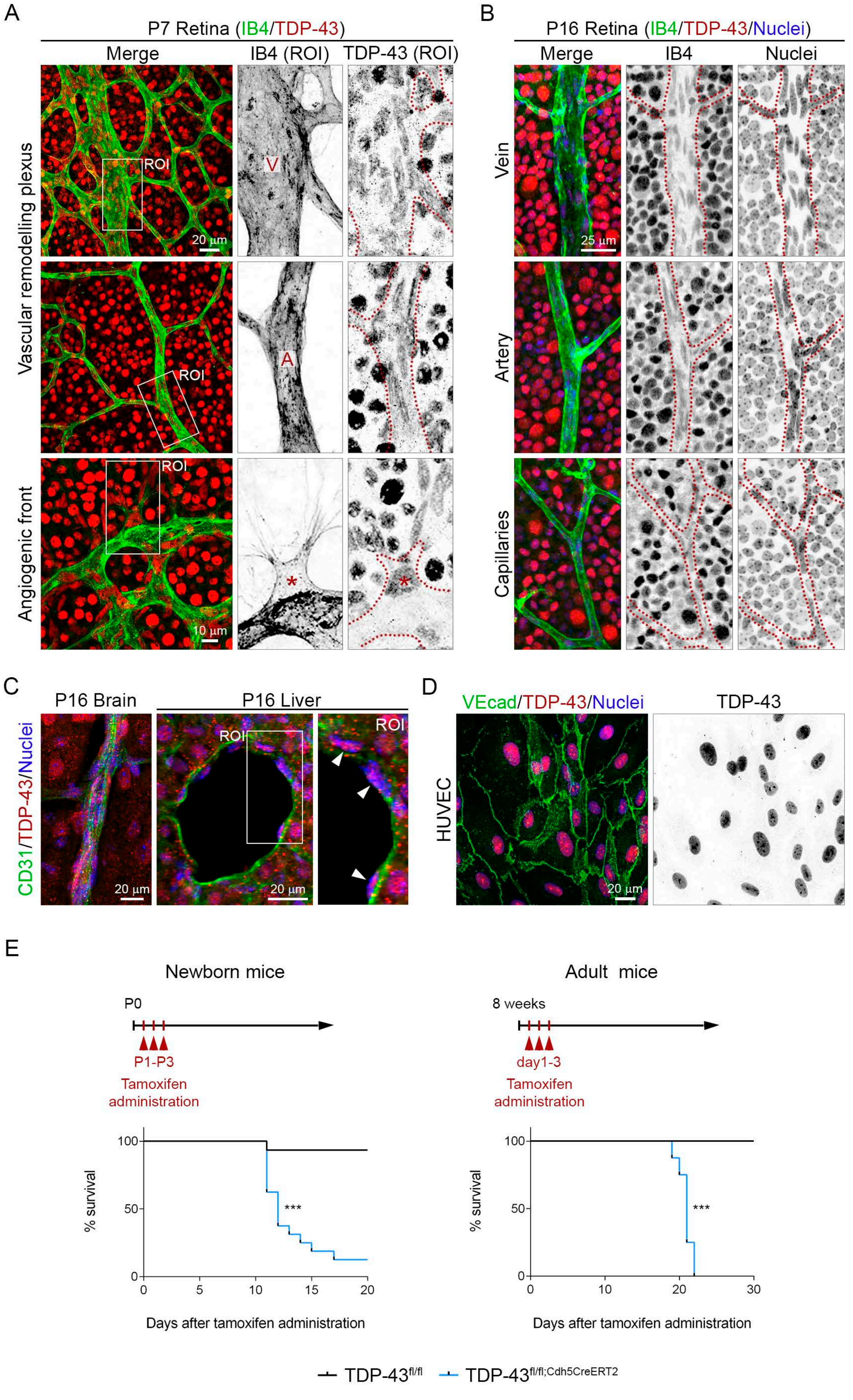
A
P7 Retina (IB4/TDP-43)
Merge
IB4 (ROI)
TDP-43 (ROI)
Vascular remodelling plexus
Angiogenic front
ROI
V
A
20 µm
10 µm
B
P16 Retina (IB4/TDP-43/Nuclei)
Merge
IB4
Nuclei
Vein
Artery
Capillaries
25 µm
C
P16 Brain
P16 Liver
CD31/TDP-43/Nuclei
ROI
20 µm
D
VEcad/TDP-43/Nuclei
TDP-43
HUVEC
20 µm
E
Newborn mice
P0
P1-P3
Tamoxifen administration
Adult mice
8 weeks
day1-3
Tamoxifen administration
% survival
***
Days after tamoxifen administration
TDP-43fl/fl
TDP-43fl/fl;Cdh5CreERT2

Supplemental Figure 1. Expression of TDP-43 in the vasculature. Confocal high-magnification images of P7 (**A**) and P16 (**B**) wild-type mouse retinas stained for IB4 (green), TDP-43 (red) and and Hoechst (nuclei, blue). White rectangles indicate the magnified ROIs. Dotted lines highlight the endothelial border of the blood vessels. Veins (V), arteries (A) and sprouting ECs (asterisks) are indicated. (**C**) Confocal high-magnification images of P16 control mouse brain and liver sections stained for CD31 (green), TDP-43 (red) and Hoechst (nuclei, blue). White rectangle indicate the magnified ROI. (**D**) Confocal high-magnification images of HUVECs stained for VEcad (green), TDP-43 (red) and Hoechst (nuclei, blue). (**E**) Kaplan-Meier survival curve for newborn and adult $TDP\text{-}43^{fl/fl}$ and $TDP\text{-}43^{fl/fl;Cdh5CreERT2}$ mice. p values (***p<0.001). Log-rank (Mantel-Cox) test.

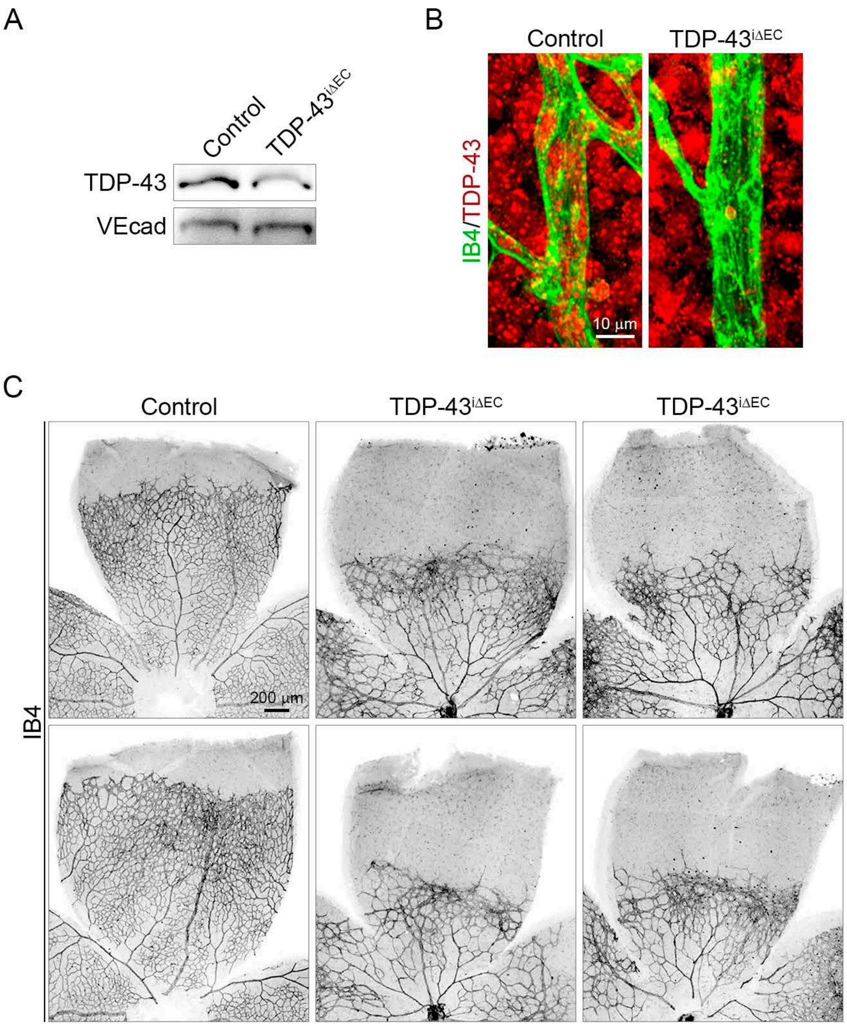
A
Control
TDP-43iΔEC
TDP-43
VEcad
B
Control
TDP-43iΔEC
IB4/TDP-43
10 µm
C
Control
TDP-43iΔEC
TDP-43iΔEC
IB4
200 µm

Supplemental Figure 2. ***Cdh5(PAC)-Cre^{ERT2}*****-mediated deletion of** ***Tdp-43*** **gene.** (**A**) Western blot analysis of TDP-43 in lung lysates from P6 control and *TDP-43$^{i\Delta EC}$* mice three days after tamoxifen administration. VEcad was used as a loading control. (**B**) Confocal high-magnification images of P7 control and *TDP-43$^{i\Delta EC}$* retinas stained for IB4 (green) and TDP-43 (red). (**C**) Confocal high-magnification images of P7 control and *TDP-43$^{i\Delta EC}$* retinas stained for IB4.

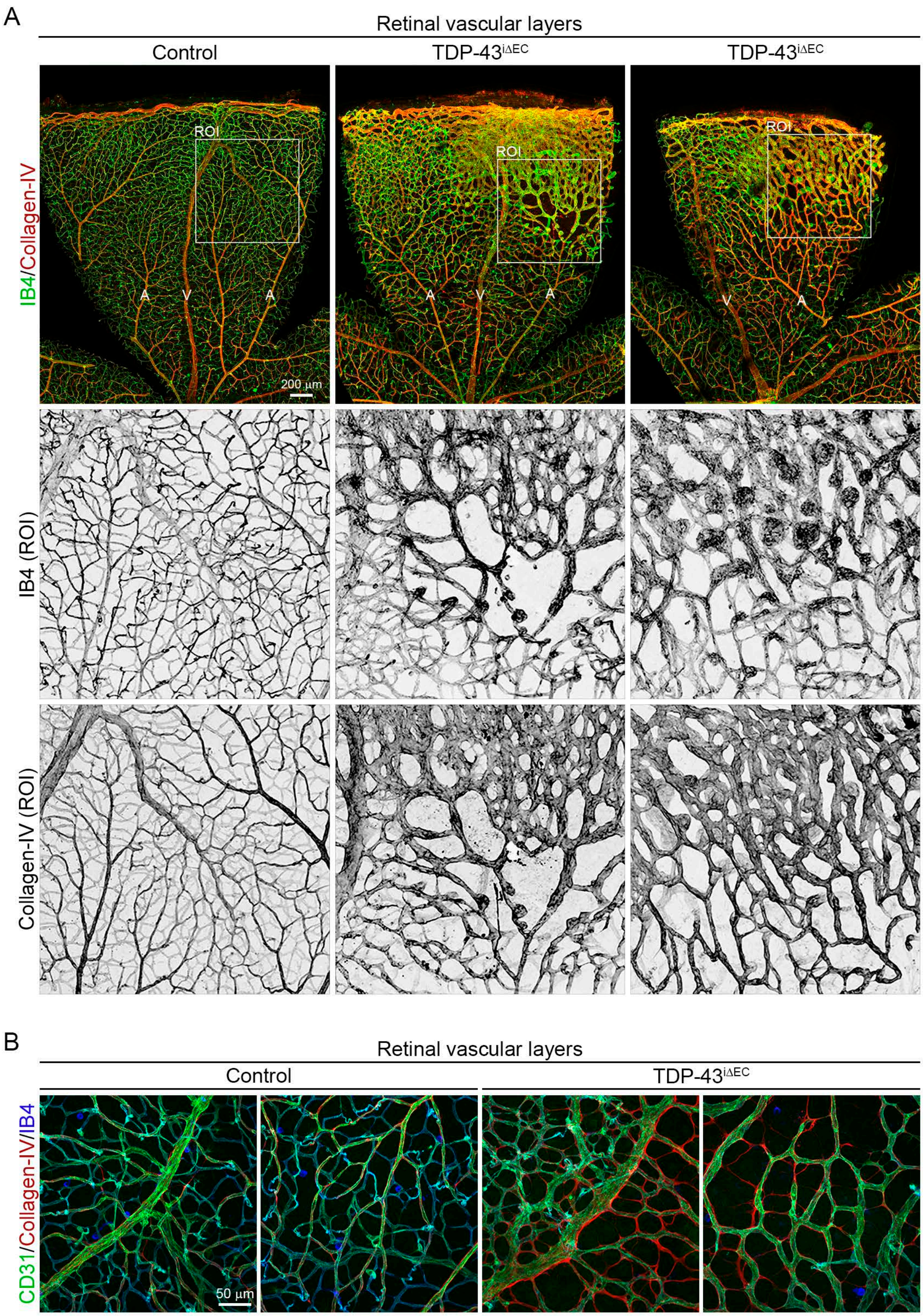
A
Retinal vascular layers
Control
TDP-43iΔEC
TDP-43iΔEC
IB4/Collagen-IV
ROI
A
V
200 µm
IB4 (ROI)
Collagen-IV (ROI)
B
Retinal vascular layers
Control
TDP-43iΔEC
CD31/Collagen-IV/IB4
50 µm

Supplemental Figure 3. Vascular malformation in *TDP-43*$^{i\Delta EC}$ mice. (**A**) Confocal high-magnification images of P16 control and *TDP-43*$^{i\Delta EC}$ retinas stained for IB4 (green) and Collagen-IV (red). White squares indicate the magnified ROIs. (**B**) Confocal high-magnification images of P16 control and *TDP-43*$^{i\Delta EC}$ retinas stained for CD31 (green), Collagen-IV (red) and IB4 (blue).

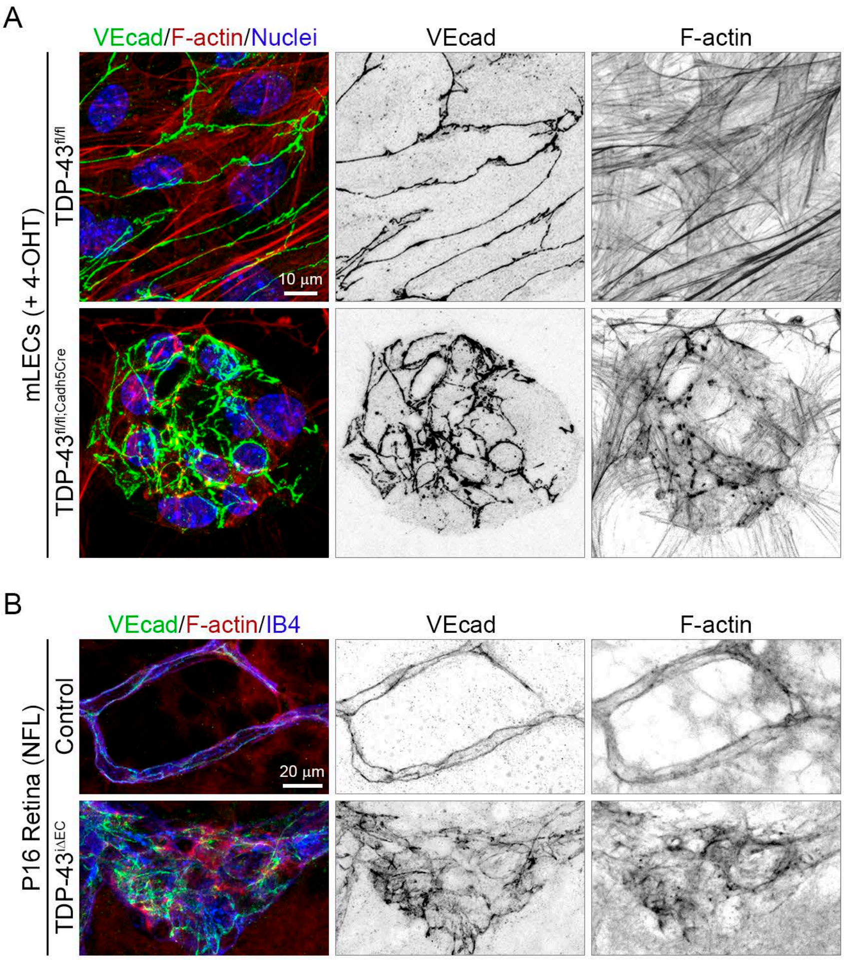
A
VEcad/F-actin/Nuclei
VEcad
F-actin
mLECs (+ 4-OHT)
TDP-43fl/fl
TDP-43fl/fl;Cadh5Cre
10 µm
B
VEcad/F-actin/IB4
VEcad
F-actin
P16 Retina (NFL)
Control
TDP-43iΔEC
20 µm

Supplemental Figure 4. Loss of TDP-43 disrupts the endothelial adherens junctions. (**A**) Confocal high magnification images of *TDP-43*$^{fl/fl}$ and *TDP-43*$^{fl/fl;Cadh5Cre}$ mLECs stained for VEcad (green), F-actin (red) and Hoechst (nuclei, blue). (**B**) Confocal high-magnification images of P16 control and *TDP-43*$^{i\Delta EC}$ retinas stained for VEcad (green), F-actin (red) and IB4 (blue).

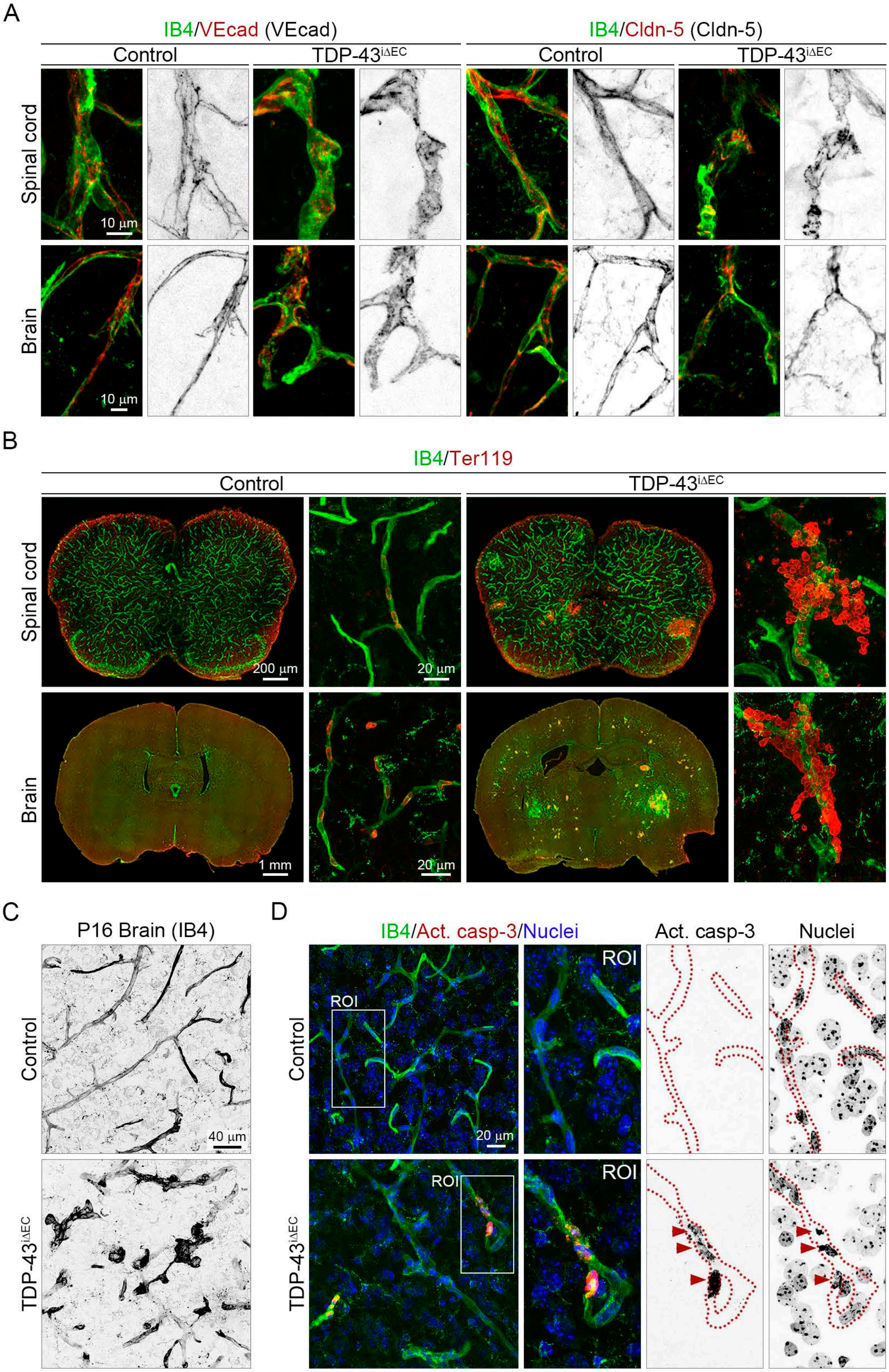
A
IB4/VEcad (VEcad)
IB4/Cldn-5 (Cldn-5)
Control
TDP-43iΔEC
Spinal cord
Brain
10 μm
B
IB4/Ter119
200 μm
20 μm
1 mm
C
P16 Brain (IB4)
40 μm
D
IB4/Act. casp-3/Nuclei
Act. casp-3
Nuclei
ROI

Supplemental Figure 5. Loss of TDP-43 disrupts the blood-CNS barrier and triggers vascular degeneration in the CNS. (**A**) Confocal high-magnification images of P16 control and *TDP-43*$^{i\Delta EC}$ spinal cord and brain sections stained for IB4 (green), VEcad (red) and Cldn-5 (red). (**B**) Confocal images of P16 control and *TDP-43*$^{i\Delta EC}$ spinal cord and brain sections stained for IB4 (green) and Ter119 (red), visualizing RBC leakage in *TDP-43*$^{i\Delta EC}$ vessels. (**C**) Confocal high-magnification images of P16 control and *TDP-43*$^{i\Delta EC}$ brain sections stained for IB4. Note the abnormal morphology of *TDP-43*$^{i\Delta EC}$ brain vessels. (**D**) Confocal high-magnification images of P16 control and *TDP-43*$^{i\Delta EC}$ brain sections stained for IB4 (green), Act. casp-3 (red) and Hoechst (nuclei, blue). White rectangles indicate magnified ROIs. Arrowheads indicate Act. casp-3 positive ECs. Dotted lines highlight the endothelial border of brain capillaries.

A

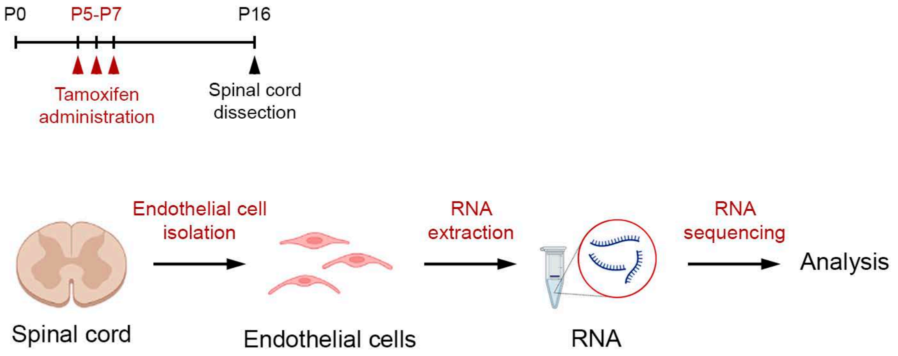
P0
P5-P7
P16
Tamoxifen administration
Spinal cord dissection
Endothelial cell isolation
RNA extraction
RNA sequencing
Analysis
Spinal cord
Endothelial cells
RNA

B

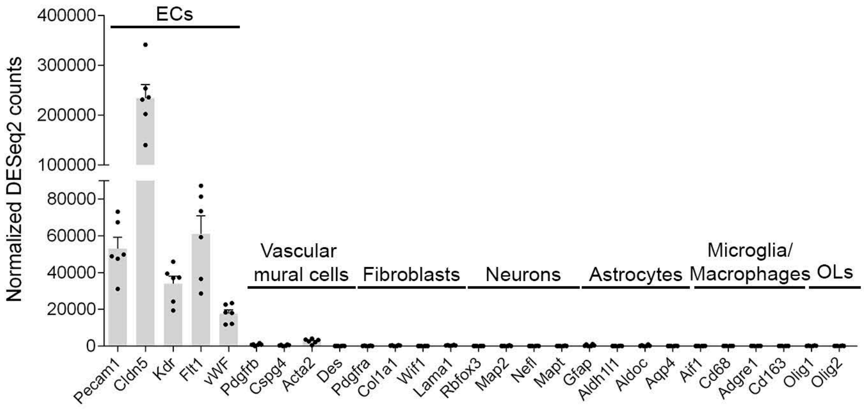
Normalized DESeq2 counts
ECs
Vascular mural cells
Fibroblasts
Neurons
Astrocytes
Microglia/ Macrophages
OLs
Pecam1
Cldn5
Kdr
Flt1
vWF
Pdgfrb
Cspg4
Acta2
Des
Pdgfra
Col1a1
Wif1
Lama1
Rbfox3
Map2
Nefl
Mapt
Gfap
Aldh1l1
Aldoc
Aqp4
Aif1
Cd68
Adgre1
Cd163
Olig1
Olig2

C

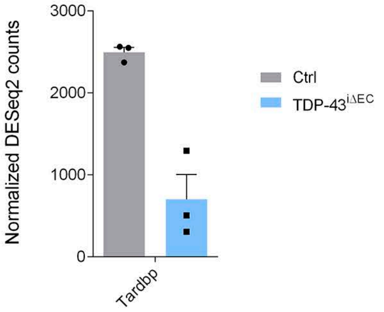
Normalized DESeq2 counts
Tardbp
Ctrl
TDP-43^iΔEC

Supplemental Figure 6. RNA sequencing analysis. (**A**) Diagram depicting the experiment schedule for ECs isolation and RNA sequencing analysis from spinal cords of P16 control and *TDP-43*$^{i\Delta EC}$. (**B**) Detailed comparison of expression levels for ECs, vascular mural cells, fibroblasts, neurons, astrocytes, microglia/macrophages and oligodendrocytes (OLs) canonical genes. (**C**) Detailed comparison of expression levels for *Tardbp* gene in ECs isolated from P16 control and *TDP-43*$^{i\Delta EC}$ spinal cords.

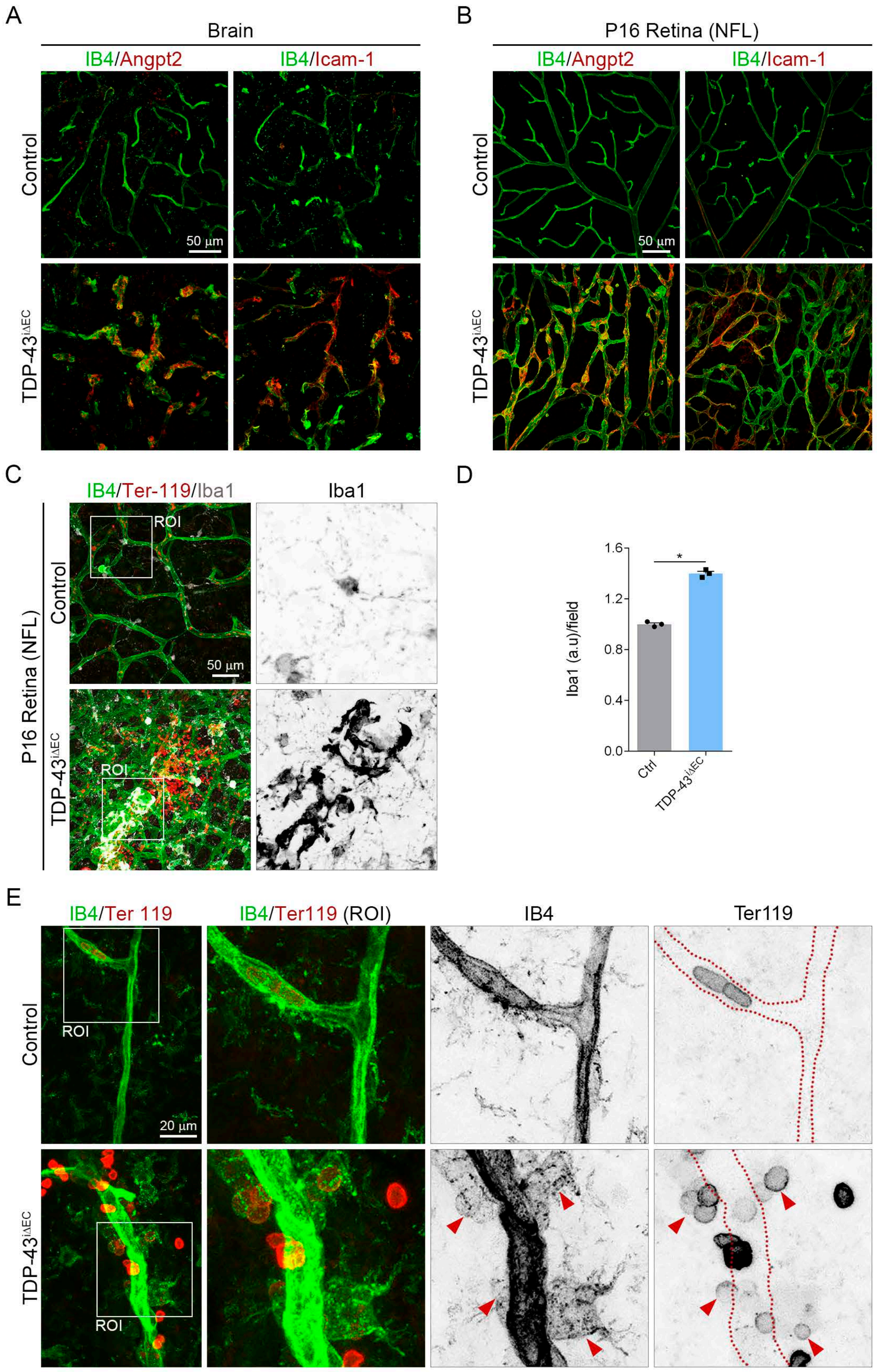
A
Brain
IB4/Angpt2
IB4/Icam-1
Control
TDP-43iΔEC
50 µm
B
P16 Retina (NFL)
IB4/Angpt2
IB4/Icam-1
Control
TDP-43iΔEC
50 µm
C
P16 Retina (NFL)
IB4/Ter-119/Iba1
Iba1
Control
TDP-43iΔEC
ROI
50 µm
D
Iba1 (a.u)/field
1.6
1.2
0.8
0.4
0.0
*
Ctrl
TDP-43iΔEC
E
IB4/Ter 119
IB4/Ter119 (ROI)
IB4
Ter119
Control
TDP-43iΔEC
ROI
20 µm

Supplemental Figure 7. Inflammation in *TDP-43*$^{i\Delta EC}$ brain and retina. Confocal high-magnification images of IB4 (green), Angpt2 (red) and Icam-1 (red) in P16 brain (**A**) and retina (**B**). (**C**) Confocal high-magnification images of P16 control and *TDP-43*$^{i\Delta EC}$ retinas stained for IB4 (green), Ter119 (red) and Iba1 (white). White squares indicate the magnified ROIs. Note the enhanced Iba1 staining in *TDP-43*$^{i\Delta EC}$ samples and the morphological differences between Iba1+ cells from *TDP-43*$^{i\Delta EC}$ and control retinas. (**D**) Quantification of Iba1 fluorescence intensity per field in control and *TDP-43*$^{i\Delta EC}$ retinas as indicated. Error bars, s.e.m. p values (*$p<0.05$). Mann-Whitney U test. (**E**) Confocal high-magnification images of P7 control and *TDP-43*$^{i\Delta EC}$ brain sections stained for IB4 (green) and Ter119 (red). White squares indicate the magnified ROIs. Note the presence of extravasated RBCs engulfed by IB4-positive macrophages (arrowheads) in *TDP-43*$^{i\Delta EC}$ mice. Dotted lines highlight the endothelial border of the blood vessels.

Supplemental Table 1. List of commercial antibodies and reagents.

Antigen/staining reagent	Source, number	Company	Dilution
β-Catenin (D10A8)	Rabbit, 8480	Cell Signaling	1:100
CD31 (MEC 13.3)	Rat, 553370	BD Pharmingen™	-
Claudin 5	Rabbit, 34-1600	ThermoFisher Scientific	1:100
Claudin 5 (4C3C2)	Mouse, 35-2500	ThermoFisher Scientific	1:100
Cleaved caspase-3	Rabbit, 9661	Cell Signaling	1:200
Collagen-IV	Rabbit, 2150-1470	Bio-Rad	1:200
Fibronectin	Rabbit, F3648	Sigma Aldrich	1:100
GAPDH	Mouse, MAB374	Chemicon	1:1000
GFAP	Rabbit, Z0334	Dako	1:250
Hoechst 33342	H3570	Invitrogen	1: 5000
Icam-1	Rabbit, ab222736	Abcam	1:100
Iba1	Rabbit, 019-19741	Wako	1:1000
Ki67	Rabbit, ab16667	Abcam	1:100
mAngiopoietin-2	Sheep, AF7186	R&D Systems	1:100
Plvap	Rat, 550563	BD Pharmingen™	1:100
TDP-43	Rabbit, 10782-2-AP	Proteintech	1:100 (IF mice), 1:500 (IF HUVECs), 1:5000 (WB)
Ter119	Rat, MAB1125	R&D Systems	1:100
α-Tubulin (DM1A)	Mouse, CP06	Calbiochem	1:1000
VE-cadherin (16B1)	Mouse, 14-1449-82	ThermoFisher Scientific	1:100
VE-cadherin	Rat, 14-1442-85	Invitrogen	1:100
ERG Alexa Fluor-647	Rabbit, ab196149	Abcam	1:100
Paxillin	Mouse, 610051	BD Pharmingen™	1:100
Phalloidin Alexa Fluor-633	A2284	Invitrogen	1:500 (IF HUVECs), 1:100 (IF mice)
Isolectin-B4 Alexa Fluor-488	I21411	Invitrogen	1:150
Mouse Alexa Fluor-546	Donkey, A10036	Invitrogen	1: 500
Rabbit Alexa Fluor-488	Chicken, A21441	Invitrogen	1: 500
Rabbit Alexa Fluor-546	Donkey, A10040	Invitrogen	1:500

Rabbit Alexa Fluor-633	Goat, A21070	Invitrogen	1:500
Rat Alexa Fluor-546	Goat, A11081	Invitrogen	1:500
Rat Alexa Fluor-633	Goat, A21094	Invitrogen	1:500
Sheep Alexa Fluor-546	Donkey, A21098	Invitrogen	1:500
APC Rat IgG2a κ Isotype Control	553932	BD Pharmingen™	1:1000
APC Rat Anti-mouse CD31 (Clone: MEC 13.3)	561815	BD Pharmingen™	1:1000
PE-Cy™7 Mouse IgG1 κ Isotype Control	557646	BD Pharmingen™	1:1000
PE-Cy™7 Rat Anti-mouse CD45 (Clone: 30-F11)	552848	BD Pharmingen™	1:1000
PE-Cy™7 Rat Anti-mouse TER-119/Erythroid cells	557853	BD Pharmingen™	1:1000
