# Supplementary figures and images for: Endothelial TDP-43 controls sprouting angiogenesis and vascular barrier integrity, and its deletion triggers neuroinflammation
Source: JCI Insight. 2024 Feb 1;9(5):e177819. doi: 10.1172/jci.insight.177819 (PMC11143933; doi:10.1172/jci.insight.177819)

Full unedited gels for figure 2B

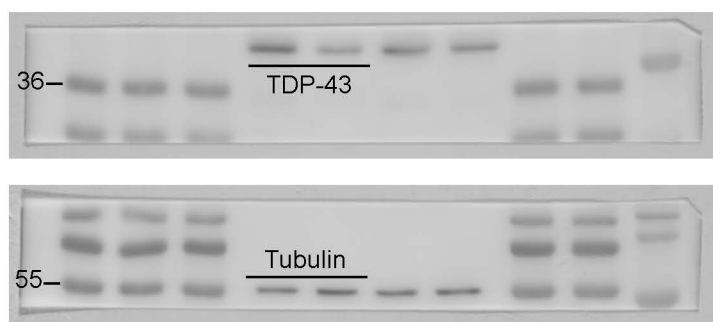

Full unedited gels for figure 2E

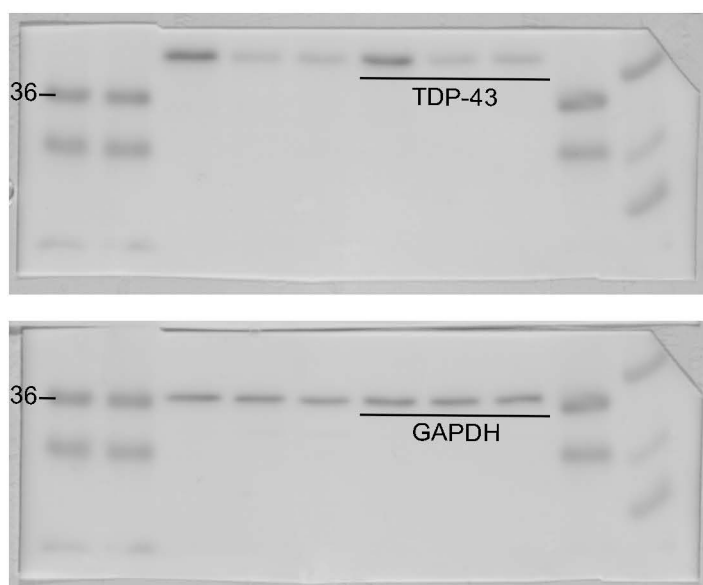

Supplement: Unedited blot and gel images [file jciinsight-9-177819-s069.pdf]
